# Infectious virus shedding duration reflects secretory IgA antibody response latency after SARS-CoV-2 infection

**DOI:** 10.1073/pnas.2314808120

**Published:** 2023-12-22

**Authors:** Sho Miyamoto, Takara Nishiyama, Akira Ueno, Hyeongki Park, Takayuki Kanno, Naotoshi Nakamura, Seiya Ozono, Kazuyuki Aihara, Kenichiro Takahashi, Yuuki Tsuchihashi, Masahiro Ishikane, Takeshi Arashiro, Shinji Saito, Akira Ainai, Yuichiro Hirata, Shun Iida, Harutaka Katano, Minoru Tobiume, Kenzo Tokunaga, Tsuguto Fujimoto, Michiyo Suzuki, Maki Nagashima, Hidenori Nakagawa, Masashi Narita, Yasuyuki Kato, Hidetoshi Igari, Kaori Fujita, Tatsuo Kato, Kazutoshi Hiyama, Keisuke Shindou, Takuya Adachi, Kazuaki Fukushima, Fukumi Nakamura-Uchiyama, Ryota Hase, Yukihiro Yoshimura, Masaya Yamato, Yasuhiro Nozaki, Norio Ohmagari, Motoi Suzuki, Tomoya Saito, Shingo Iwami, Tadaki Suzuki

**Affiliations:** ^a^Department of Pathology, National Institute of Infectious Diseases, Tokyo 162-8640, Japan; ^b^Interdisciplinary Biology Laboratory, Division of Natural Science, Graduate School of Science, Nagoya University, Aichi 464-8602, Japan; ^c^International Research Center for Neurointelligence, The University of Tokyo Institutes for Advanced Study, The University of Tokyo, Tokyo 113-0033, Japan; ^d^Center for Emergency Preparedness and Response, National Institute of Infectious Diseases, Tokyo 162-8640, Japan; ^e^Center for surveillance, Immunization, and Epidemiologic Research, National Institute of Infectious Diseases, Tokyo 162-8640, Japan; ^f^Center for Field Epidemic Intelligence, Research and Professional Development, National Institute of Infectious Diseases, Tokyo 162-8640, Japan; ^g^Disease Control and Prevention Center, National Center for Global Health and Medicine, Tokyo 162-8655, Japan; ^h^Department of Infectious Diseases, Osaka City General Hospital, Osaka 534-0021, Japan; ^i^Division of Infectious Diseases, Department of Internal Medicine, Okinawa Prefectural Nanbu Medical Center and Children’s Medical Center, Okinawa 901-1193, Japan; ^j^Department of Infectious Diseases, International University of Health and Welfare Narita Hospital, Chiba 286-0124, Japan; ^k^Department of Infection Control, Chiba University Hospital, Chiba, Japan; ^l^Department of Respiratory Medicine, National Hospital Organization Okinawa National Hospital, Okinawa 901-2214, Japan; ^m^Department of Chest Disease, National Hospital Organization Nagara Medical Center, Gifu 502-8558, Japan; ^n^Department of Infectious Disease, National Hospital Organization Fukuoka-Higashi Medical Center, Fukuoka 811-3195, Japan; ^o^Department of Pediatrics, Hirakata City Hospital, Osaka 573-1013, Japan; ^p^Department of Infectious Diseases, Tokyo Metropolitan Toshima Hospital, Tokyo 173-0015, Japan; ^q^Department of Infectious Disease, Tokyo Metropolitan Cancer and Infectious Diseases Center Komagome Hospital, Tokyo 113-8677, Japan; ^r^Department of Infectious Diseases, Tokyo Metropolitan Bokutoh Hospital, Tokyo 130-8575, Japan; ^s^Department of Infectious Diseases, Japanese Red Cross Narita Hospital, Chiba 286-8523, Japan; ^t^Division of Infectious Disease, Yokohama Municipal Citizen’s Hospital, Kanagawa 221-0855, Japan; ^u^Department of General Internal Medicine and Infectious Diseases, Rinku General Medical Center 598-8577, Osaka, Japan; ^v^Department of Respiratory Medicine, Tokoname City Hospital, Aichi 479-8510, Japan; ^w^Institute of Mathematics for Industry, Kyushu University, Fukuoka 819-0395, Japan; ^x^Institute for the Advanced Study of Human Biology, Kyoto University, Kyoto 606-8501, Japan; ^y^Interdisciplinary Theoretical and Mathematical Sciences Program, RIKEN, Saitama 351-0198, Japan; ^z^NEXT-Ganken Program, Japanese Foundation for Cancer Research, Tokyo 135-8550, Japan; ^aa^Science Groove Inc., Fukuoka 810-0041, Japan

**Keywords:** mucosal secretory IgA, infectious virus shedding duration, human-to-human transmission, COVID-19 vaccine, prior-infection

## Abstract

Infectious virus shedding from individuals infected with severe acute respiratory syndrome-coronavirus 2 (SARS-CoV-2) is used to estimate human-to-human transmission risk. Control of SARS-CoV-2 transmission requires identifying the immune correlates that protect infectious virus shedding. Mucosal immunity prevents infection by SARS-CoV-2, which replicates in the respiratory epithelium and spreads rapidly to other hosts. However, whether mucosal immunity prevents the shedding of the infectious virus in SARS-CoV-2-infected individuals is unknown. We examined the relationship between viral RNA shedding dynamics, duration of infectious virus shedding, and mucosal antibody responses during SARS-CoV-2 infection. Anti-spike secretory IgA antibodies (S-IgA) reduced viral RNA load and infectivity more than anti-spike IgG/IgA antibodies in infected nasopharyngeal samples. Compared with the IgG/IgA response, the anti-spike S-IgA post-infection responses affected the viral RNA shedding dynamics and predicted the duration of infectious virus shedding regardless of the immune history. These findings highlight the importance of anti-spike S-IgA responses in individuals infected with SARS-CoV-2 for preventing infectious virus shedding and SARS-CoV-2 transmission. Developing medical countermeasures to shorten S-IgA response time may help control human-to-human transmission of SARS-CoV-2 infection and prevent future respiratory virus pandemics.

## Significance

Understanding the factors that influence human-to-human transmission of SARS-CoV-2 (severe acute respiratory syndrome-coronavirus-2) is crucial for controlling the pandemic. Additionally, identifying the immune pathways that regulate infectious virus shedding from individuals infected with SARS-CoV-2 is essential for estimating the inter-individual virus transmission risk, as infectious viral shedding from SARS-CoV-2-infected individuals is considered a useful surrogate for estimating the risk of human-to-human transmission. This study investigated the impact of mucosal antibody responses on the prevention of infectious virus shedding in individuals infected with SARS-CoV-2. Our findings establish the clinical significance of mucosal antibodies on prevention of respiratory viruses’ transmission, which would provide the impetus for the development of technologies to control future pandemics caused by respiratory viruses.

Understanding the factors that influence human-to-human transmission of respiratory viral infections, such as coronavirus disease-19 (COVID-19), is critical for epidemic control, which necessitates research on the relationship between infectious viral shedding and immunity in individuals infected with severe acute respiratory syndrome-coronavirus-2 (SARS-CoV-2) (1, 2). As SARS-CoV-2 infects the respiratory epithelium and is transmitted via respiratory droplets and aerosols (3, 4), it is postulated that secretory IgA antibodies (S-IgA) distributed in the respiratory mucosa play a crucial role in preventing SARS-CoV-2 infection (5–7). S-IgA produced by plasma cells in the lamina propria of mucosal tissues binds to the poly-Ig receptor (pIgR) expressed on the basolateral side of mucosal epithelial cells and is transported to the mucosal surface. On the mucosal surface, the extracellular portion of pIgR is cleaved and incorporated into the IgA structure as a secretory component (SC), referred to as S-IgA (8). S-IgA is multivalent, and unlike IgG, S-IgA exists as dimers, trimers, and tetramers (9). These multimeric S-IgAs present higher coherency and avidity to respiratory virus antigens, contributing to higher and broader neutralizing activity (9–13). In addition, S-IgA not only provides immediate immunity by eliminating respiratory viruses before they cross the mucosal barrier (14) but is also anticipated to decrease the infectivity of viruses shed by infected individuals (5).

Mucosal IgA to SARS-CoV-2 can be found in the saliva, nasal secretions, tears, tracheal-bronchial secretions, and breast milk of individuals infected with SARS-CoV-2 (15–21), and anti-spike S-IgA has been reported to be induced in the saliva of patients with a history of SARS-CoV-2 infection after intramuscular vaccination with an mRNA vaccine (22). In addition, individuals with high levels of IgA against the SARS-CoV-2 ancestral strain spike antigen in the nasal mucosa show decreased risk of Omicron-breakthrough infection (23), indicating that IgA in the nasal mucosa has a highly cross-protective effect against infection with SARS-CoV-2 variants. However, respiratory viruses, such as SARS-CoV-2, replicate extremely rapidly on the respiratory epithelium and disseminate to subsequent hosts before the adaptive immune response is completely activated (24). Therefore, how the presence of S-IgA in the upper respiratory tract affects the viral shedding dynamics or the duration of infectious viral shedding in SARS-CoV-2–infected individuals is not well understood.

Here, we measured the viral RNA load, viral titer, and mucosal antibody levels, including IgG, IgA, and S-IgA levels, in longitudinally collected nasopharyngeal samples from Omicron-infected individuals and generated high-resolution trajectories of viral RNA shedding in the upper respiratory tract to examine the relationship between viral RNA shedding dynamics, infectious virus shedding duration, and mucosal antibody responses. We then proposed an approach for evaluating mucosal antibody responses linked to duration of infectious virus shedding and determined the mucosal antibodies that contributed most significantly to the control of infectious virus shedding. Our observations will help in developing a mucosal immunity-targeting vaccine, which will be critical for preventing inter-human transmission and controlling pandemics by respiratory viruses in the future.

## Results

### Anti-Spike S-IgA Is the Most Effective Nasal Antibody in Reducing Viral RNA Load and Infectivity in Nasopharyngeal Samples.

**Fig. 1.**
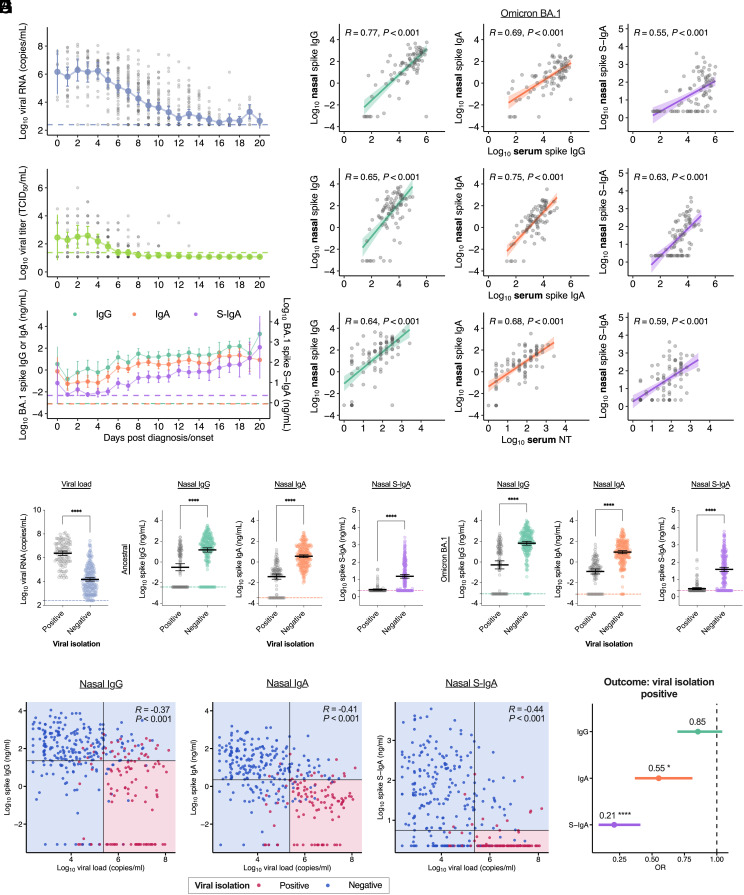
Prediction of virus isolation by nasal anti-spike S-IgA titer against Omicron variants: (*A*) Dynamics of viral load and anti-spike antibodies in nasal swabs in the days following diagnosis or onset of COVID-19 in SARS-CoV-2 Omicron-infected cases. Mean ± 95% CI of viral RNA load (*Top*), infectious viral titer (*Middle*), and anti-spike antibody titers against the Omicron BA.1 variant (*Bottom*) are shown. Dotted lines indicate the detection limits. For the viral RNA load and viral titer, individual data points are displayed. (*B*) Correlation between nasal antibody titers and serum antibody titers for symptomatic cases with matching collection dates against the Omicron BA.1 variant. Regression lines with 95% CIs, Pearson correlation R values, and *P* values are shown. (*C*) Comparison of viral RNA load in samples that are positive or negative for viral isolation among PCR-positive samples. (*D*) Comparison of nasal IgG, IgA, and S-IgA against the ancestral strain and BA.1 variant spike in samples that are positive or negative for viral isolation. Titers between the positive and negative samples were compared using unpaired *t* tests. (*E*–*G*) Verification of the accuracy of cut-off values estimated using logistic regressions. The areas and dots represent the predictive value and viral isolation test outcomes, respectively. Vertical and horizontal lines indicate cut-off values as shown in *SI Appendix*, Fig. S1*D*. Pearson correlation R values, and *P* values are shown. (*H*) Logistic regression analysis of virus isolation-test positive samples, considering nasal anti-BA.1 spike antibody titers. Forest plot showing OR and 95% CI. Statistical significance: ns, not significant; *P < 0.05; **P < 0.01; ***P < 0.001; ****P < 0.0001.

First, we measured the viral RNA load, viral titer, and nasal IgG/IgA/S-IgA titers against the spike protein of the ancestral strain and the Omicron BA.1 variant in 590 nasopharyngeal samples obtained from 122 Omicron-variant-infected individuals who enrolled in the Omicron first few hundred (FF100) cohort studies conducted from December 2021 to January 2022 in Japan. The characteristics of the patients included in this study are summarized in *SI Appendix*, Table S1. Longitudinal evaluation of these measured values over time after onset or diagnosis (whichever came first) revealed that the viral RNA load and viral titer decreased over time, whereas nasal antibodies to Omicron BA.1 spike increased over time (Fig. 1*A* and *SI Appendix*, Table S1). We also measured serum neutralization titers (NT) and serum anti-spike IgG/IgA titers against the ancestral strain and Omicron BA.1 in 86 samples obtained from 86 of the 122 cases (*SI Appendix*, Table S1). To evaluate the correlation between nasal and serum antibodies, we compared nasal antibody titers with serum antibody titers collected within ±1 d of nasopharyngeal specimen collection. In instances where multiple pairs of corresponding serum and nasopharyngeal specimens were available for a single case, the specimen pair was evaluated using the latest collection date. Nasal anti-spike IgG, IgA, and S-IgA against the Omicron BA.1 variant correlated positively with serum anti-spike IgG, IgA, and serum NT in all combinations (Fig. 1*B*). Notably, serum and nasal anti-spike IgA and nasal anti-spike S-IgA were less strongly associated with vaccination status than serum and nasal anti-spike IgG (*SI Appendix*, Fig. S1*A*). Additionally, the correlation between nasal S-IgA and serum antibodies was weaker than that between nasal IgG and IgA and serum antibodies (Fig. 1*B*). Considering the specificity of the S-IgA secreted on mucosal surfaces (8), these results suggested that nasal S-IgA may be an appropriate proxy for mucosal-specific antibody responses.

Next, we compared the viral RNA load in virus-isolation-positive and viral-negative samples among PCR-positive nasopharyngeal samples (n = 418, Fig. 1*C*). Nasal IgG/IgA/S-IgA to the ancestral strain or Omicron BA.1 spike antigens of the isolation-negative samples were significantly higher than those of the isolation-positive samples (Fig. 1*D*). Furthermore, a significant negative correlation was observed between the viral RNA load and the amount of each mucosal anti- BA.1 spike antibody, such as IgG, IgA, or S-IgA, with the virus isolation-positive specimens having high viral RNA load and low titers of each antibody (Fig. 1 *E*–*G*). Viral RNA load and mucosal S-IgA titer have shown the most significant negative correlation coefficient among the three antibody isotypes (R = −0.44, Fig. 1*G*). These results suggested that nasal anti-spike antibodies contributed to reduction of the viral RNA load and infectivity in the nasal mucosa.

A logistic regression model was then applied to estimate the relationship between the viral RNA load and the probability of a positive virus isolation test in nasopharyngeal samples, resulting in a high area under the receiver operating characteristic curve (ROC-AUC) of 0.915 (*SI Appendix*, Fig. S1*B*). Using the criterion of the point on the ROC curve with the highest Youden Index, we calculated a cut-off value for the viral RNA load of 5.364 (log_10_ copy/mL) with specificity (84.5%) and sensitivity (85.2%) for predicting the virus isolation test (*SI Appendix*, Fig. S1*E*). This also indicated that several nasopharyngeal samples contained relatively high viral RNA loads and tested negative for viral isolation. To evaluate the contribution of each antibody isotype in decreasing the infectivity of the nasopharyngeal samples, we applied a logistic regression model to estimate the relationship between anti-BA.1 spike IgG, IgA, or S-IgA and the probability of isolation-positive test results in nasopharyngeal samples, achieving high ROC-AUC for IgG, IgA, or S-IgA (0.817, 0.883, and 0.857, respectively) (*SI Appendix*, Fig. S1*C*). Using the same criterion for the ROC curve, we calculated a cut-off value for anti-BA.1 spike IgG, IgA, or S-IgA of 22.54, 2.22, or 5.60 (ng/mL) for predicting the result of the virus isolation test, respectively (*SI Appendix*, Fig. S1*D*), revealing that the cut-off values for anti-BA.1 spike IgA/S-IgA were lower than that for anti-BA.1 spike IgG.

Using combinations of the cut-off values, nasopharyngeal samples obtained from Omicron-variant-infected individuals with viral RNA more than the viral RNA threshold and antibodies less than each threshold of nasal anti-BA.1 spike IgG, IgA, or S-IgA were predicted to contain the infectious virus with high specificities of 95.3%, 94.8%, or 94.0% and sensitivities of 64.0%, 74.1%, and 76.9%, respectively (Fig. 1 *E*–*G* and *SI Appendix*, Fig. S1*E*). To compare the contribution of each antibody isotype (IgG, IgA, and S-IgA) in decreasing the infectivity of the nasopharyngeal samples, we applied logistic regression analysis with the isolation-positive test results as the outcome variable and anti-BA.1 spike mucosal antibodies as the predictor variables (*SI Appendix*, Fig. S1*F*). Notably, S-IgA was a significant predictor (*P* < 0.0001) of positive virus isolation, with an odds ratio (OR) of 0.21 (95% CI, 0.09 to 0.41) (Fig. 1*H*).

To compensate for the potential impact of sampling efficiency of nasopharyngeal mucus, we measured total IgG or IgA in nasopharyngeal swab fluid and the proportion of anti-spike IgG/IgA/S-IgA antibodies in the total amount of IgG/IgA antibodies in the nasopharyngeal swab fluid was calculated. These normalized anti-spike antibody titers showed the same increasing trend as the non-normalized anti-spike antibody titers (*SI Appendix*, Fig. S2 *A* and *B*). In terms of predictive accuracy for virus isolation, the AUCs were as high as the non-normalized anti-spike antibody titers (*SI Appendix*, Figs. S1*C* and S2*C*). Additionally, normalized anti-spike S-IgA antibody titers among mucosal antibodies were also shown to be most strongly associated with viral isolation test results (*SI Appendix*, Fig. S2*D*). Moreover, both normalized anti-spike IgA and S-IgA demonstrated more pronounced negative correlations with viral RNA load (*SI Appendix*, Fig. S2*E*) and showed higher sensitivity and specificity against virus isolation outcome than non-normalized anti-spike IgA and S-IgA (*SI Appendix*, Fig. S2*F*). These results suggested that the proportion of anti-spike S-IgA in nasal IgA also predicts the result of the virus isolation test.

These findings indicated that nasal anti-spike S-IgA is the nasal antibody most strongly associated with decreased viral RNA load and infectivity in the nasopharyngeal mucosa. However, whether and how the reduction in viral RNA load and infectivity caused by mucosal antibodies affects the duration of infectious virus shedding were not clear.

### Peak Viral Load Is a Major Determinant of the Duration of Infectious Virus Shedding.

**Fig. 2.**
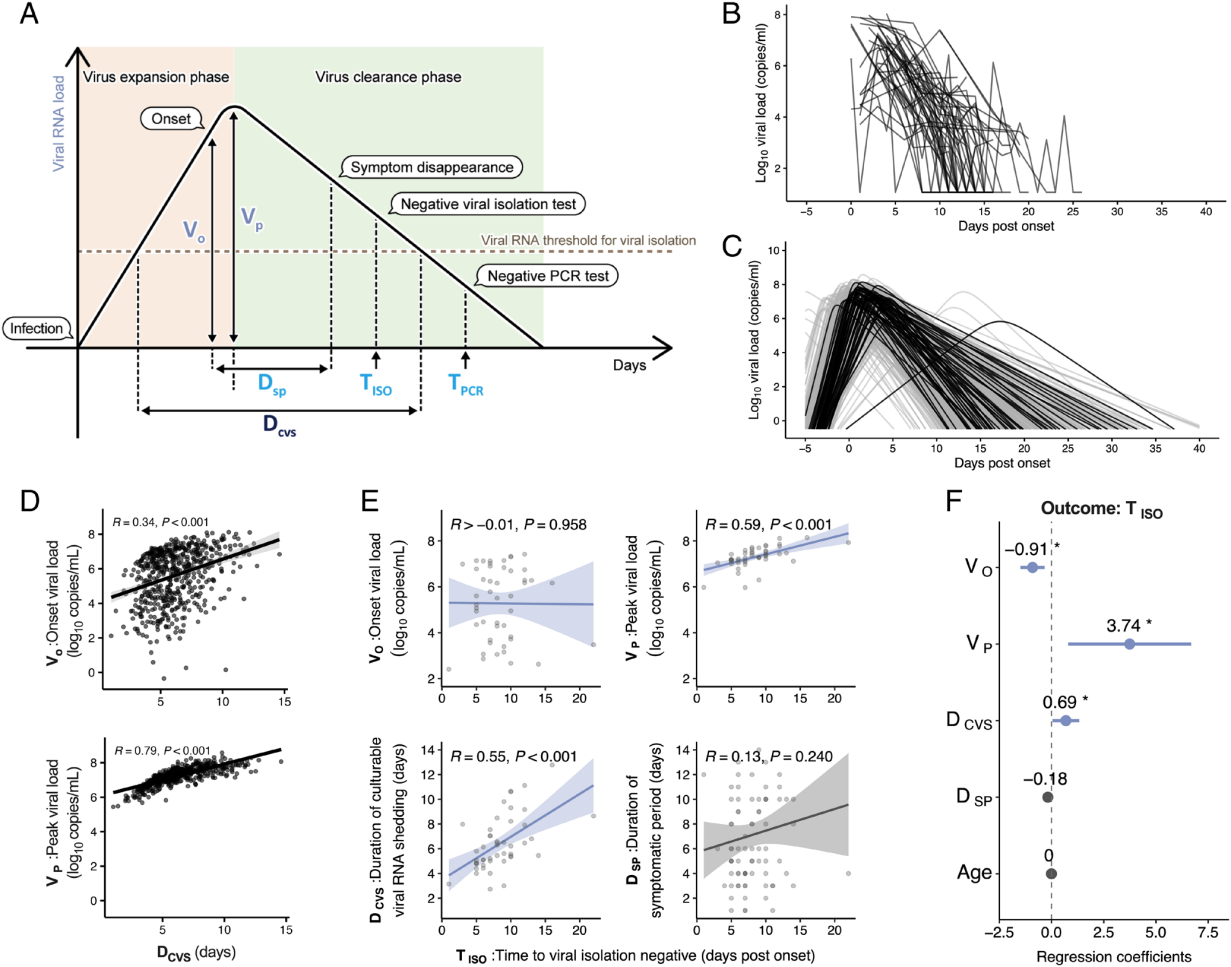
Quantification and characterization of the dynamics of viral RNA shedding: (*A*) Illustration of parameters (Table 1) derived from virus dynamics related to time and amount. (*B*) The measured values of individual viral RNA dynamics in the respiratory samples obtained from the FF100 cohort. (*C*) The reconstructed individual-level viral RNA dynamics based on mathematical modeling. Black and gray colors correspond to the viral loads calculated from the FF100 and NBA cohorts, respectively. (*D*) Correlation between the features extracted from the reconstructed individual-level viral RNA dynamics. Regression lines with 95% CIs, Pearson correlation R values, and *P* values are shown. (*E*) Association between the infectious virus shedding period and the features extracted from the reconstructed individual-level viral RNA dynamics. Correlation between post-onset time to viral isolation negative ( $T_{\mathrm{ISO}}$ ) and onset viral load ( $V_{O}$ ), peak viral load ( $V_{P}$ ), duration of culturable viral RNA shedding ( $D_{\mathrm{CVS}}$ ), and duration of symptomatic period ( $D_{\mathrm{SP}}$ ) are shown, respectively. (*F*) Multiple regression analysis of the infectious virus shedding period ( $T_{\mathrm{ISO}}$ ), including the features extracted from the reconstructed viral RNA dynamics. Forest plot showing regression coefficients and 95% CI. Statistical significance: ns, not significant; *P < 0.05; **P < 0.01; ***P < 0.001; ****P < 0.0001.

To determine the factors associated with the duration of viral shedding in individuals infected with Omicron, we first characterized the viral RNA shedding dynamics in each case. We used a previously developed mathematical model to describe the SARS-CoV-2 shedding dynamics (*SI Appendix*, Eqs. **1** and **2**) and reconstruct the best-fit dynamics of each individual infected with the Omicron considering inter-patient heterogeneity in viral shedding. For this analysis, we obtained similar viral RNA load data for symptomatic individuals infected with Omicron from the National Basketball Association (NBA) cohort (521 cases) (25) and symptomatic cases from the FF100 cohort with three or more viral RNA-positive nasopharyngeal specimens (51 cases), and used these datasets for our analysis of viral RNA shedding dynamics, as described in *SI Appendix*, Fig. S3*A*. In total, 572 symptomatic patients were included in the analysis. The individual-level model fits from the FF100 cohort are described in *SI Appendix*, Fig. S4. The definition of each feature constituting the viral RNA shedding dynamics is shown in Fig. 2*A* and Table 1, and the estimated fixed and individual parameters are summarized in *SI Appendix*, Tables S2 and S3. Please note that our data from the FF100 cohort mainly pertained to the late phase of infection (after symptom onset) described in Fig. 2*B*, whereas data from the NBA cohort pertained to the early phase (before symptom onset); therefore, the datasets complemented each other. Plots of viral shedding dynamics for each case reconstructed from the hybrid data of both cohorts showed that the characteristics of viral shedding dynamics for the cases from the FF100 and NBA cohorts matched well, suggesting the adequacy of the model composed of two different cohorts (Fig. 2*C* and *SI Appendix*, Table S4).

**Table 1.** Definition of features describing viral shedding and the dynamics of the mucosal antibody response

Feature name	Figure label	Explanation	Source
Duration of symptomatic period	$D_{SP}$	Number of days during the observation period in which any clinical symptoms related to COVID-19 were observed.	Measured value FF100 cohort
IgG response latency	$L_{IgG}$	The day when the anti-spike IgG titer first exceeded the threshold for infectious virus suppression during the observation period.	Measured value FF100 cohort
IgA response latency	$L_{IgA}$	The day when the anti-spike IgA titer first exceeded the threshold for infectious virus suppression during the observation period.	Measured value FF100 cohort
S-IgA response latency	$L_{S-IgA}$	The day when the anti-spike S-IgA titer first exceeded the threshold for infectious virus suppression during the observation period.	Measured value FF100 cohort
Time to viral isolation negative	$T_{ISO}$	The first day of a negative viral culture test after the last positive viral culture test with nasopharyngeal samples during the observation period. Used as a measured value for the infectious virus shedding duration.	Measured value FF100 cohort
Time to PCR negative	$T_{PCR}$	The first day of a negative PCR test after the last positive PCR test with nasopharyngeal samples during the observation period, or the last day of the observation period in which the patient passed the serial testing of the criteria to end isolation (whichever came first). Used as a measured value for the viral RNA shedding duration.	Measured value FF100 cohort
Duration of culturable viral RNA load shedding	$D_{CVS}$	Period in which viral RNA loads above the viral RNA load threshold with a high probability of being culturable are detected in the viral RNA dynamics of each case constructed in the model.	Model estimate
Onset viral load	$V_{O}$	The amount of viral RNA at the onset of symptoms determined in the viral RNA dynamics of each case constructed in the model.	Model estimate
Peak viral load	$V_{P}$	Peak amount of viral RNA determined in viral RNA dynamics for each case constructed in the model.	Model estimate

To identify the predictors of the duration of viral RNA shedding based on the dynamic features of viral shedding, we evaluated the relationship among the model-estimated values of the reconstructed dynamics, onset viral load ( $V_{O}$ ), peak viral load ( $V_{P}$ ), and duration of culturable viral RNA shedding ( $D_{\mathrm{CVS}}$ ). Notably, $V_{P}$ vs. $D_{\mathrm{CVS}}$ showed a higher positive correlation than Vo vs. $D_{\mathrm{CVS}}$ , suggesting that the strength of the association between viral RNA load (amount of viral RNA; Vo and $V_{P}$ ) and duration of viral RNA shedding ( $D_{\mathrm{CVS}}$ ) was not constant, but changed over time after the infection. Additionally, $V_{P}$ , rather than $V_{O}$ , was a potential predictor of the duration of viral RNA shedding (Fig. 2*D*).

Next, to evaluate the relationship between the estimated values of viral RNA shedding dynamics and the measured values regarding the duration of infectious virus shedding of the FF100 cohort cases, we compared the model-estimated values ( $V_{O}$ , $V_{P}$ , and $D_{\mathrm{CVS}}$ ) with time to becoming viral isolation-negative after onset ( $T_{\mathrm{ISO}}$ ), a proxy indicator for the infectious virus shedding duration. Among the estimated values for viral RNA load, $V_{P}$ , but not $V_{O}$ , correlated positively with $T_{\mathrm{ISO}}$ (Fig. 2*E*). Additionally, the viral RNA shedding duration-related value, $D_{\mathrm{CVS}}$ , correlated positively with $T_{\mathrm{ISO}}$ (Fig. 2*E*). In contrast, the duration of symptomatic period ( $D_{\mathrm{SP}}$ ) did not show any significant correlation with $T_{\mathrm{ISO}}$ (Fig. 2*E*). These results indicated that the peak viral load and duration of viral RNA shedding are important for determining the duration of infectious virus shedding. Furthermore, multiple regression analysis considering the covariates for $T_{\mathrm{ISO}}$ revealed that $V_{P}$ , rather than $D_{\mathrm{CVS}}$ , was associated with $T_{\mathrm{ISO}}$ with a high effect size (Fig. 2*F*). This suggested that among the features of viral RNA shedding dynamics, the peak viral load reflected the duration of infectious virus shedding.

Notably, the measured time to becoming PCR-negative after onset ( $T_{\mathrm{PCR}}$ ) showed a strong positive correlation with $D_{\mathrm{CVS}}$ among the model-estimated values (*SI Appendix*, Fig. S3*B*). Additionally, multiple regression analysis considering the covariates for $T_{\mathrm{PCR}}$ showed that $D_{\mathrm{CVS}}$ was associated with $T_{\mathrm{PCR}}$ (*SI Appendix*, Fig. S3*C*). This highlights the differences between the durations of infectious viral shedding and viral RNA shedding, and confirms the accuracy of the model-estimated value regarding the duration of viral RNA shedding, $D_{\mathrm{CVS}}$ . Collectively, these results suggested that evaluation of the peak viral load was useful for predicting the duration of infectious virus shedding.

### Correlation of Nasal Anti-Spike S-IgA Response Latency with Duration of Infectious Virus Shedding.

**Fig. 3.**
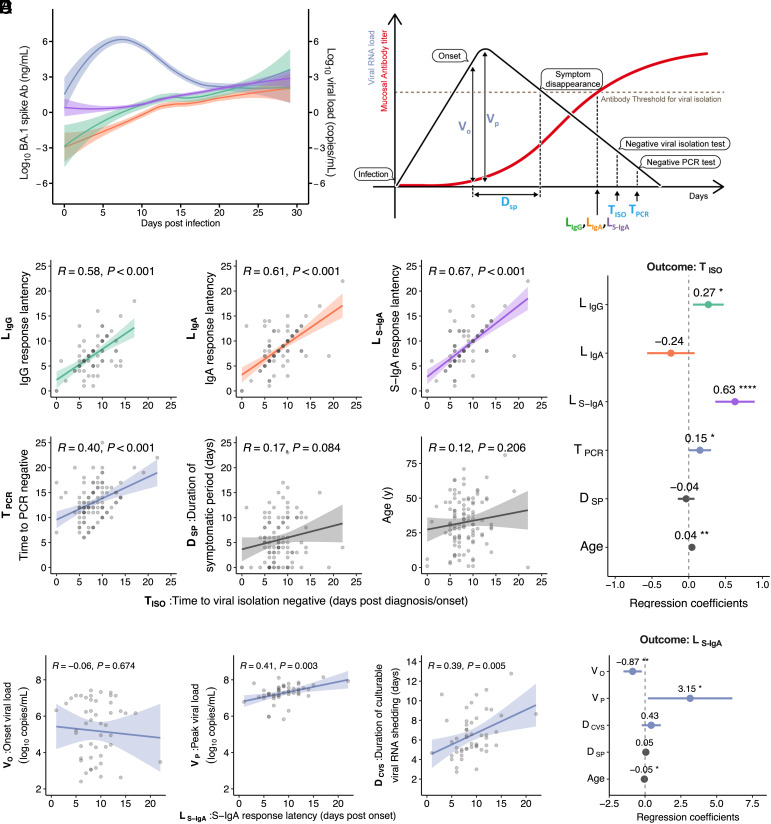
Relationship between nasal anti-spike S-IgA response latency and dynamics of virus shedding: (*A*) Time-series patterns of viral RNA load (blue), IgG (green), IgA (orange), and S-IgA (pink) for total patients are shown. Lines indicate cross-sectional averages from each group, with shading representing 95% CI and colored accordingly. (*B*) Illustration of time-related parameters derived from S-IgA dynamics. Details of the parameters are shown in Table 1. (*C*) Correlations between time to viral isolation negative after diagnosis/onset and each of the following: Antibody response latencies after diagnosis/onset ( $L_{\mathrm{IgG}}$ , $L_{\mathrm{IgA}}$ , and $L_{S-IgA}$ ), time to PCR negative after diagnosis/onset, and duration of symptomatic period are shown, along with its multiple regression analysis (*D*). Regression lines with 95% CIs, Pearson correlation R values, and *P* values are shown. Forest plot showing regression coefficients and 95% CI. (*E*) Correlations between post-onset S-IgA response latency ( $L_{S-IgA}$ ) and each of the following parameters: onset viral load ( $V_{O}$ ), peak viral load ( $V_{P}$ ), and duration of culturable viral RNA shedding ( $D_{\mathrm{CVS}}$ ). (*F*) Multiple regression analysis of $L_{S-IgA}$ , including the features extracted from the reconstructed viral RNA dynamics. Statistical significance: ns, not significant; *P < 0.05; **P < 0.01; ***P < 0.001; ****P < 0.0001.

Next, we evaluated the relationship between the duration of viral shedding and the dynamics of mucosal antibodies that control viral infectivity in the upper respiratory tract. To profile nasal anti-spike antibody responses, we used longitudinal anti-spike IgG, IgA, and S-IgA in nasopharyngeal samples obtained from the same patients in the FF100 cohort, as described above (Fig. 2). The time-dependent patterns of nasal IgG, IgA, and S-IgA levels against the spike of the Omicron BA.1 variant after infection are described in Fig. 3*A*, along with the viral RNA load. Anti-spike IgG, IgA, and S-IgA levels increased over time with similar dynamics for approximately 1 mo after infection. Considering these patterns of steadily increasing mucosal antibodies, we suggested that the latency period of each antibody response after infection, which is the time required to induce the mucosal antibody levels necessary to suppress infectious viruses in nasopharyngeal samples, could be used as a surrogate indicator of mucosal antibody responses, contributing to the prevention of infectious virus shedding in individuals infected with Omicron (Fig. 3*B*). To determine the immune correlates for the duration of infectious virus shedding, the time at which the IgG, IgA, and S-IgA levels exceeded the threshold for suppressing viral infectivity (cut-off value for viral isolation-negative) in each case [IgG response latency ( $L_{\mathrm{IgG}}$ ), IgA response latency ( $L_{\mathrm{IgA}}$ ), and S-IgA response latency ( $L_{S-IgA}$ )] was obtained (Fig. 3*B* and Table 1). The $L_{\mathrm{IgG}}$ , $L_{\mathrm{IgA}}$ , and $L_{S-IgA}$ levels correlated strongly and positively with the duration of infectious virus shedding ( $T_{\mathrm{ISO}}$ ) (Fig. 3*C*). The $T_{\mathrm{ISO}}$ also correlated positively with the measured duration of viral RNA shedding (i.e., $T_{\mathrm{PCR}}$ ), but not with $D_{\mathrm{SP}}$ and age (Fig. 3*C*). Multiple regression analysis considering covariates for $T_{\mathrm{ISO}}$ revealed that among mucosal antibody responses and viral RNA shedding duration, $L_{S-IgA}$ was associated most strongly with $T_{\mathrm{ISO}}$ (Fig. 3*D*). Additionally, we did not find any differences in sex or symptom onset for $L_{S-IgA}$ and $T_{\mathrm{ISO}}$ among the groups (*SI Appendix*, Fig. S5 *A* and *B*). These results suggested that virus-specific S-IgA response latency acts as an immune correlate of the duration of infectious virus shedding, which is consistent with the results shown in Fig. 1*H*.

Additionally, we evaluated the relationship between $L_{S-IgA}$ and model-estimated values describing viral RNA shedding dynamics. Among the model-estimated values related to viral load, $V_{P}$ , but not $V_{O}$ , correlated positively with $L_{S-IgA}$ (Fig. 3*E*). Additionally, the viral RNA shedding duration-related value, $D_{\mathrm{CVS}}$ , showed similar positive correlation with $L_{S-IgA}$ (Fig. 3*E*). Consistent with the results of multiple regression analysis for $T_{\mathrm{ISO}}$ (Fig. 2*F*), multiple regression analysis considering covariates for $L_{S-IgA}$ revealed that $V_{P}$ was strongly associated with $L_{S-IgA}$ (Fig. 3*F*). This indicated that the peak viral load may reflect the mucosal antibody response after infection and influence the duration of infectious viral shedding. However, $L_{\mathrm{IgG}}$ and $L_{\mathrm{IgA}}$ exhibited weaker correlations with $V_{P}$ than with $L_{S-IgA}$ (*SI Appendix*, Fig. S5 *C* and *E*) and no association with $V_{P}$ in the multiple regression analysis (*SI Appendix*, Fig. S5 *D* and *F*). Collectively, these results suggested that mucosal S-IgA response latency significantly influenced the duration of infectious virus shedding and correlated with the prevention of infectious virus shedding.

### Prior Infection History Was Associated with Short Nasal Anti-Spike S-IgA Response Latency and Duration of Short Viral Shedding.

Finally, we investigated whether a patient’s immune history (infection and vaccination) altered the mucosal antibody response latency and duration of viral shedding because a short S-IgA response latency group ( $L_{S-IgA}$ ≤ 5) had a significantly higher proportion of SARS-CoV-2 infection history (*SI Appendix*, Table S5). We stratified all cases, including asymptomatic cases from the FF100 cohort, into three groups according to their immune history: an unvaccinated group with no history of SARS-CoV-2 infection (naive), a group with a history of one to three vaccinations but no prior-SARS-CoV-2 infection (vaccination-only), and a group with prior-SARS-CoV-2 infection history, regardless of vaccination history (prior infection) (*SI Appendix*, Table S6).

**Fig. 4.**
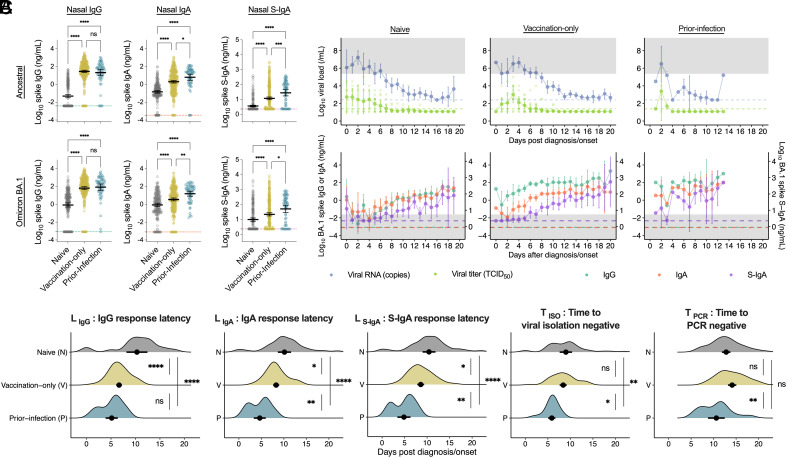
Effect of immune history on S-IgA response latency and viral shedding duration: (*A*) Comparison of nasal IgG, IgA, and S-IgA against the ancestral strain and BA.1 variant spike in groups with different immune histories: no vaccination or SARS-CoV-2 infection history (naive), a history of 1 to 3 vaccinations but no prior-SARS-CoV-2 infection (vaccination-only), or prior-SARS-CoV-2 infection history regardless of vaccination history (prior-infection). Mean ± 95% CI is shown. Antibody titers between the groups were compared using Tukey’s test. Dotted lines indicate the detection limits. (*B*) Dynamics of viral load and anti-spike antibodies in nasal swabs in the days after diagnosis or onset in each immune history group. Mean ± 95% CI of viral RNA load, infectious viral titer, and anti-BA.1 spike antibody titers are shown. Dotted lines indicate the detection limits. Individual data are displayed in the viral tier. Gray regions indicate regions above the viral RNA threshold or below the S-IgA threshold. (*C*) Comparison of post-diagnosis/onset parameters obtained from the FF100 cohort among the exposure histories. Mean ± 95% CI is shown. Significance was measured using one-way ANOVA corrected using Tukey’s test. Statistical significance: ns, not significant; *P < 0.05; **P < 0.01; ***P < 0.001; ****P < 0.0001.

The levels of nasal anti-spike IgG, IgA, and S-IgA in the ancestral strain and BA.1 variant were lowest in the naive group and highest in the prior infection group (Fig. 4*A* and *SI Appendix*, Table S6). Longitudinal evaluation of viral RNA load, viral titer, and nasal antibody titers over time after onset or diagnosis for each immune history group revealed that infectious viral titers were negative when viral RNA loads and S-IgA crossed each threshold for virus isolation, as shown in Fig. 1*G* (Fig. 4*B* and *SI Appendix*, Table S6). Additionally, the vaccination-only group showed a more rapid onset of IgG responses than IgA or S-IgA responses, whereas the prior infection group showed rapid onset of all antibody responses and short antibody response latency for all antibodies (Fig. 4*B*).

We compared the differences in the duration after diagnosis/onset regarding viral shedding and mucosal antibody responses, including the durations of infectious virus shedding ( $T_{\mathrm{ISO}}$ ) and viral RNA shedding ( $T_{\mathrm{PCR}}$ ), $L_{\mathrm{IgG}}$ , $L_{\mathrm{IgA}}$ , and $L_{S-IgA}$ among the naive, vaccination-only, and prior-infection groups (Fig. 4*C*). Notably, the prior infection history group showed the lowest $L_{\mathrm{IgA}},\;L_{S-IgA}$ , and $T_{\mathrm{ISO}}$ levels among the three groups. However, the vaccination-only group showed markedly shorter $L_{\mathrm{IgG}}$ than the naive group but did not differ from the naive group in $T_{\mathrm{ISO}}$ or $T_{\mathrm{PCR}}$ (Fig. 4*C*). Additionally, a comparison of the vaccine-only and naive groups using model estimates showed that all features describing viral RNA shedding dynamics were comparable between the two groups (*SI Appendix*, Fig. S6 *A* and *B*). The differences between $T_{\mathrm{ISO}}$ and $L_{\mathrm{IgA}}$ , or $T_{\mathrm{ISO}}$ and $L_{S-IgA}$ were approximately 0 d for any of the immune history groups and were comparable among these groups, suggesting a more direct effect on $T_{\mathrm{ISO}}$ for $L_{\mathrm{IgA}}$ and $L_{S-IgA}$ than for the immuno history (*SI Appendix*, Fig. S7*A*). Meanwhile, a divergence between $T_{\mathrm{ISO}}$ and $L_{\mathrm{IgG}}$ was noted in the naive and vaccination only groups. Notably, only $L_{S-IgA}$ among the antibody response latencies showed a significantly higher positive correlation with $T_{\mathrm{ISO}}$ across all immuno history groups (*SI Appendix*, Fig. S7 *B–D*). To evaluate the $L_{S-IgA}$ and $T_{\mathrm{ISO}}$ of the hybrid immunity group (Hybrid) having exposure history to both vaccination and prior infection with a small number of cases (n = 8), hierarchical Bayesian modeling was employed, considering the probability distribution of all cases. Estimations showed that $L_{\mathrm{IgG}}$ , $L_{\mathrm{IgA}}$ , $L_{S-IgA}$ , $T_{\mathrm{ISO}}$ , and $T_{\mathrm{PCR}}$ were comparable between the Prior-infection only (prior infection history without vaccination) and Hybrid groups (*SI Appendix*, Fig. S8*A*). It was also confirmed that $L_{\mathrm{IgA}}$ and $L_{S-IgA}$ tend to coincide with $T_{\mathrm{ISO}}$ in any group (*SI Appendix*, Fig. S8*B*). These findings demonstrated that the virus-specific S-IgA response latency robustly reflected the duration of infectious virus shedding, even with different immune histories. Collectively, these results suggested that a history of prior infection with SARS-CoV-2, but not vaccination, significantly enhanced rapid and efficient nasal anti-spike S-IgA responses that shortened the duration of infectious virus shedding.

## Discussion

In this study, we demonstrated that induction of antiviral S-IgA is associated with reduced viral RNA load and infectivity in the nasal mucosa. Furthermore, our study showed that the promptness of the antiviral S-IgA response in the nasal mucosa after infection correlated with viral RNA shedding dynamics and predicted the duration of infectious virus shedding, strongly suggesting that S-IgA functions as a mechanistic immunological correlate for preventing infectious virus shedding.

Antibodies in the nasal mucosa are primarily composed of the IgA isotype (9, 10), while the IgG isotype in the nasal mucosa is considered a blood spillover. In this study, nasal anti-spike IgG levels correlated highly with serum anti-spike IgG levels. Nasal IgA contains the monomeric IgA form not bound to SC, and the monomeric IgA in the nasal mucosa is also considered a blood spillover (9). The antibodies exuded from the blood may not accurately reflect the physiological state of mucosal antibody responses because they may be actively exuded as a result of the physical stimuli encountered during sample collection. However, as SC-bound S-IgA antibodies are physiologically present only in the mucosa and not in the blood (8), the influence of spillover antibodies from the blood to the mucosa can be ignored by specifically measuring nasal S-IgA in nasopharyngeal specimens. The nasal antiviral S-IgA response measured in this study demonstrated a low correlation with serum antiviral antibodies, suggesting that the S-IgA response is an appropriate proxy for mucosa-specific antibody response. Recent reports showed that individuals with high levels of SARS-CoV-2 spike-specific mucosal IgA and S-IgA are less likely to experience breakthrough infection with the Omicron variant, suggesting that mucosal S-IgA plays a substantial role in cross-protection against SARS-CoV-2 variants (23, 26). Furthermore, nasal antibodies after breakthrough infection with Omicron BA.1 or BA.2 virus possess neutralizing activity against BA.5 and Delta viruses (27), indicating that mucosal S-IgA has high cross-protective activity against SARS-CoV-2 variant infections.

Antibodies present in the mucosa, such as S-IgA, neutralize viruses in the saliva and nasal secretions of infected individuals and reduce reinfection by preventing infectious virus shedding (5, 9, 10). However, the clinical significance of mucosal antibodies in preventing viral shedding in respiratory viral infections has not been established because of the lack of appropriately designed clinical studies. Nasopharyngeal viral loads in COVID-19 patients are strongly associated with the human-to-human transmission of SARS-CoV-2 infection (28). Additionally, positive viral culture results in the upper respiratory tract specimens from COVID-19 patients have been used as indicators of the presence of infectious viruses shed from infected individuals (29). Therefore, the dynamics of viral RNA and shedding should be understood to determine the risk of contracting reinfections. This study demonstrated that individuals infected with Omicron variants with shorter S-IgA response latencies had shorter duration of infectious virus shedding and lower peak viral load, regardless of vaccination status. S-IgA secreted on the mucosa prior to viral exposure contributes to protection from infection; however, even in case the amount of S-IgA is insufficient to protect against infection, the rapid induction of sufficient amounts of S-IgA to suppress the infectivity of progeny viruses after infection may be important in reducing the risk of reinfection in infected individuals. A report from the human challenge trial showed that the amount of the virus needed to infect more than 50% of humans with SARS-CoV-2 was a 10 median tissue culture infectious dose (TCID_50_) (30). Given that the detection limit for the virus isolation test was 24 TCID_50_/mL in our study, the anti-spike S-IgA antibody titer (6.5 ng/mL) is comparable to the antibody titer required to suppress the viral titer (10 TCID_50_) to establish infection. Furthermore, the current study revealed that infected individuals with the shortest S-IgA response latency had prior history of infection, highlighting the importance of antigen stimulation of the mucosal route obtained by natural infection for the prompt induction of the S-IgA response after infection.

The COVID-19 pandemic highlights the need for a vaccine that can not only prevent disease but can also prevent reinfection (31), and respiratory mucosal vaccines may be capable of addressing these issues. To improve the feasibility of clinical trials of intranasal mucosal vaccines, appropriate immune correlates that can be used to predict the clinical relevance should be identified. Our study, which focused on the response latency to reach a certain level of S-IgA rather than the level of induced S-IgA, shows that the S-IgA response latency can be used as an immunological correlate to prevent infectious virus shedding from respiratory virus-infected individuals, which is precisely what is required for next-generation vaccines that will enable pandemic control (5). Such a correlation would be useful in the development of intranasal mucosal vaccines, which can induce mucosal immunity and reduce human-to-human transmission. Thus, it may be crucial not only for controlling the current SARS-CoV-2 epidemic but also for preventing the next pandemic.

### Limitations of the Study.

Despite the overall strengths of this study, it had several limitations. First, owing to the nature of observational studies using residual clinical specimens (FF100 cohort), the intervals at which nasopharyngeal specimens were collected differed between cases, and specimens were not always obtained at optimal intervals. Although our quantitative modeling approach can reconstruct the overall profiles of viral RNA dynamics at the individual level, it may prevent precise determination of the infectious virus shedding duration and S-IgA response latency for each case. Second, the small number of previously infected participants in this study may have impeded accurate evaluation of the effect of prior infection. Third, the trajectory data on nasal antiviral antibody levels should be interpreted with consideration of the infection variant, vaccination status, and time of infection; only data obtained in the present study were used, limiting the precision because of the small sample size. Finally, in COVID-19, viral culture positivity has been proposed as a surrogate for infectivity. Our findings highlight an association between anti-spike S-IgA and viral infectivity, but direct causation has not been established. Further research is required to examine the relationship among viral culture positivity, mucosal antibody responses, and the incidence of reinfection in individuals infected with SARS-CoV-2.

## Materials and Methods

### Human Participants and Sampling.

The characteristics of the human participants included in this study from the FF100 cohort study are shown in *SI Appendix*, Table S7. Demographic information, vaccination status, and information on sera and respiratory samples obtained from the FF100 cohort study were collected as part of public health activity under the Act on the Prevention of Infectious Diseases and Medical Care for Patients with Infectious Diseases. Serum and respiratory samples obtained from Omicron-variant-infected patients were collected for clinical testing during admission and submitted to the NIID for virological and serological tests as part of the public health activity. In this study, cases with a history of COVID-19 diagnosis prior to the current event or positive anti-N antibody (cut-off index > 1.0) within 5 d after onset or diagnosis were considered to have a history of SARS-CoV-2 infection based on previous reports (32). Serum antibodies were not measured prior to the study enrollment event (prior to Omicron infection) in the enrolled cases. Respiratory specimens were collected from nasopharyngeal swabs. RT-qPCR assays were performed at NIID for all respiratory samples to confirm the sample quality for genome sequencing and to quantify the viral RNA load. The infected viral strains from the FF100 cohort study were confirmed using whole-genome sequencing as described (32) and the consensus sequences were uploaded to Global Initiative on Sharing Avian Influenza Data (GISAID) (https://www.gisaid.org). Viral isolation was attempted for the RT-qPCR positive samples, as described previously (33). To evaluate the sensitivity of our virus isolation tests, we compared our T_ISO_ data with the previously published data by Boucau et al. (34). No apparent differences were observed in the efficiency of virus isolation during the same time period between the FF100 cohort in this study and the previously reported cohorts (34) involving Omicron and Delta cases (*SI Appendix*, Fig. S9*A*). Furthermore, the Unvaccinated (34), Vaccinated (34), Naive (this study), and Vaccinated-only (this study) groups all showed a similar downward trend in virus isolation rates, which differed from the trend observed in the Prior-infection (this study) group (*SI Appendix*, Fig. S9*B*). S-IgA against SARS-CoV-2 in the nasopharyngeal swabs was measured using the V-PLEX SARS-CoV-2 panel 24 (Mouse IgG) kit (Meso Scale Discovery, MD, USA). With the exception of the secondary antibody and calibrator, the assays were mostly performed according to the manufacturer’s instructions.

Additionally, similar viral load data (521 cases) were obtained from the NBA occupational health cohort (the e cohort) (25) for robust parameter estimations of our mathematical model (*SI Appendix*, Eqs. **1** and **2**). Only symptomatic patients were included and patients with fewer than three viral RNA-positive nasopharyngeal swab specimens were excluded for this mathematical modeling (we used data from 572 cases, 51 cases from the FF100 cohort and 521 cases from the NBA cohort).

## Supplementary Material

Appendix 01 (PDF)

Dataset S01 (XLSX)

## Data Availability

The characteristics of human participants included in this study are shown in *SI Appendix*, Table S7. All other data are included in the manuscript and/or supporting information.
