# Supplementary material for: Infectious virus shedding duration reflects secretory IgA antibody response latency after SARS-CoV-2 infection
Source: Proc Natl Acad Sci U S A. 2023 Dec 22;120(52):e2314808120. doi: 10.1073/pnas.2314808120 (PMC10756199; doi:10.1073/pnas.2314808120)
Supplement: Supplementary file 1 — Appendix 01 (PDF) [file pnas.2314808120.sapp.pdf]

PNAS

Supporting Information for

Infectious virus shedding duration reflects secretory IgA antibody response latency after SARS-CoV-2 infection

Sho Miyamoto, Takara Nishiyama, Akira Ueno, Hyeongki Park, Takayuki Kanno, Naotoshi Nakamura, Seiya Ozono, Kazuyuki Aihara, Kenichiro Takahashi, Yuuki Tsuchihashi, Masahiro Ishikane, Takeshi Arashiro, Shinji Saito, Akira Ainai, Yuichiro Hirata, Shun Iida, Harutaka Katano, Minoru Tobiume, Kenzo Tokunaga, Tsuguto Fujimoto, Michiyo Suzuki, Maki Nagashima, Hidenori Nakagawa, Masashi Narita, Yasuyuki Kato, Hidetoshi Igari, Kaori Fujita, Tatsuo Kato, Kazutoshi Hiyama, Keisuke Shindou, Takuya Adachi, Kazuaki Fukushima, Fukumi Nakamura-Uchiyama, Ryota Hase, Yukihiro Yoshimura, Masaya Yamato, Yasuhiro Nozaki, Norio Ohmagari, Motoi Suzuki, Tomoya Saito, Shingo Iwami, Tadaki Suzuki

Corresponding authors: Shingo Iwami and Tadaki Suzuki
Email: iwami.iblab@bio.nagoya-u.ac.jp (S.I.); tksuzuki@niid.go.jp (T.S.).

This PDF file includes:

Supporting text
Figures S1 to S9
Tables S1 to S6
SI references

Other supporting materials for this manuscript include the following:

Table S7

Supporting Information Text

Materials and Methods

Ethics statement

This study was approved by the Medical Research Ethics Committee of the National Institute of Infectious Diseases (NIID) for the use of human subjects (approval no. 1417), and the requirement for written informed consent was waived because of its retrospective design. This study was approved by the Ethics Committee of Nagoya University (approval number: hc 22-05). This study was performed on leftover clinical samples and information collected in the Omicron first few hundreds (FF100) cohort study from December 2021 to January 2022 in Japan, which was conducted as part of the public health activity led by the Ministry of Health, Labor and Welfare (MHLW) and the NIID, in cooperation with the Prevention Center within the National Center for Global Health and Medicine (NCGM/DCC), and several medical facilities (Osaka City General Hospital, Okinawa Prefectural Nanbu Medical Center & Children's Medical Center, International University of Health and Welfare Narita Hospital, Chiba University Hospital, National Hospital Organization Okinawa National Hospital, National Hospital Organization Nagara Medical Center, National Hospital Organization Fukuoka-Higashi Medical Center, Hirakata City Hospital, Tokyo Metropolitan Toshima Hospital, Tokyo Metropolitan Cancer and Infectious Diseases Center Komagome Hospital, Tokyo Metropolitan Bokutoh Hospital, Japanese Red Cross Narita Hospital, Yokohama Municipal Citizen's Hospital, Rinku General Medical Center, and Tokoname City Hospital) under the Infectious Diseases Control Law. The primary results of the FF100 cohort study were published in Japanese on the NIID website to meet the statutory requirements.

RT-qPCR and viral load data

RT-qPCR was performed as previously described (1). Nucleic acids were extracted from nasopharyngeal swabs using the MagMAX viral/pathogen nucleic acid isolation kit (Thermo Fisher Scientific, MA, USA). Quantification cycle (Cq) values (viral RNA loads) were measured using RT-qPCR with a QuantiTect Probe RT-PCR kit (Qiagen, Hilden, Germany) targeting the SARS-CoV-2 nucleoprotein (N) region via an NIID-N2 primer/probe set (2). The thermal cycling conditions were as follows: 50°C for 30 min, 95°C for 15 min, and 45 cycles of 95°C for 15 s and 60°C for 1 min. The Cq values of the samples judged negative were analyzed by substituting a Cq value of 45. Cq values were converted to viral nucleoprotein RNA copy numbers using a simple regression line (1). Viral isolation was attempted for the NIID-N2 primer/probe set-positive samples. Additionally, similar viral load data (521 cases) were obtained from the NBA occupational health cohort (the e cohort) (3) for robust parameter estimations of our mathematical model (Eq. 1-2). Only symptomatic patients were included and patients with fewer than three viral RNA-positive nasopharyngeal swab specimens were excluded for this mathematical modeling (we used data from 572 cases, 51 cases from the FF100 cohort and 521 cases from the NBA cohort).

Cells

VeroE6/TMPRSS2 cells (JCRB1819, Japanese Collection of Research Bioresources Cell Bank; Osaka, Japan) were maintained in low-glucose Dulbecco's modified Eagle's medium (DMEM) (Fujifilm, Osaka Japan) containing 10% heat-inactivated fetal bovine serum (FBS) (Biowest, Nuaillé, France), 1 mg/mL geneticin (Thermo Fisher Scientific), and 100 units/mL penicillin/streptomycin (Thermo Fisher Scientific) at 37°C in an atmosphere of 5% CO_2.

Viral isolation and titration

Viral isolation was attempted in all RT-qPCR-positive cases with available residual respiratory specimens, as described previously (1). Briefly, VeroE6/TMPRSS2 cells (JCRB1819,

JCRB Cell Bank) were seeded in 96-well flat-bottom plates and inoculated in duplicate with respiratory specimens mixed with DMEM supplemented with 2% FBS and an antibiotic-antimycotic solution (Thermo Fisher Scientific). The culture supernatant was changed to fresh medium one day post-infection (d.p.i.), and the cells were incubated at 37°C in the presence of 5% CO_2. Cytopathic effects were observed. After five days, the supernatant was collected and RT-qPCR was performed using the SARS-CoV-2 direct detection RT-qPCR kit (Takara) to confirm the propagation of SARS-CoV-2. The $TCID_{50}$ of the residual specimens was determined for all viral isolation-positive cases.

Live virus neutralization assay

The SARS-CoV-2 ancestral strain, WK-521 (lineage A, GISAID ID: EPI_ISL_408667), and the Omicron variant, TY38-873 (lineage BA.1, GISAID: EPI_ISL_7418017), were used. Live virus neutralization assays were performed as described previously (5). Briefly, serum samples were serially diluted (via two-fold dilutions starting from 1:5) in high-glucose DMEM supplemented with 2% FBS and 100 units/mL penicillin/streptomycin and were mixed with 100 $TCID_{50}$ SARS-CoV-2 viruses, followed by incubation at 37°C for 1 h. The virus-serum mixtures were placed on VeroE6/TMPRSS2 cells seeded in 96-well plates and cultured at 37°C in the presence of 5% CO_2 for five days. After culturing, the cells were fixed using 20% formalin (Fujifilm) and stained with crystal violet solution (Sigma-Aldrich, St. Louis, MO, USA). Neutralization titers were defined as the geometric mean of the reciprocal of the highest sample dilution that protected at least 50% of the cells from a cytopathic effect in 2–4 multiplicate series. As the sera from individuals were limited in quantity, this assay was performed only once. All experiments using live viruses were performed in a biosafety level 3 laboratory at the NIID.

Secretory IgA (S-IgA) titer in nasopharyngeal swabs

S-IgA against SARS-CoV-2 in the nasopharyngeal swabs was measured using the V-PLEX SARS-CoV-2 panel 24 (Mouse IgG) kit (Meso Scale Discovery, MD, USA). With the exception of the secondary antibody and calibrator, the assays were mostly performed according to the manufacturer's instructions. Briefly, the 96-well plates were blocked using MSD Blocker A for 30 min at room temperature with shaking. After washing with the washing buffer, the samples were diluted 1:50 in diluent buffer, and the MSD standard or undiluted internal MSD controls were added to the wells. After 2 h of incubation at room temperature with shaking and washing, mouse anti-human secretory IgA antibody (Millipore, HP6141) diluted to 2 g/mL in diluent buffer was added to each well. After 1 h of incubation at 37°C and a washing step, the detection antibody (MSD SULFO-TAG™ Anti-Mouse IgG Antibody) was added and incubated for 1 h at room temperature. After washing, the MSD GOLD™ Read buffer B was added and the plates were read using a MESO ™ QuickPlex SQ 120MM reader. A standard curve was established by fitting standard signals to a 4-parameter logistic model. Sample concentrations were determined from the electrochemiluminescence signals by back-fitting to the standard curve of the human monoclonal antibody S309 clone (6) in IgG1, monomeric IgA2, or secretory IgA2 form and multiplying by the dilution factor. This assay detects the secretory component common to both secretory IgA1 and IgA2, but does not distinguish between IgA1 and IgA2. The S309 antibody for the standard curve was produced by using Expi293 Expression System (Thermo Fisher Scientific) as recombinant IgG1, monomeric IgA2, or secretory IgA2 antibody (7).

Electrochemiluminescence immunoassay

To examine antibody titers, nasopharyngeal swabs and serum samples were heat-inactivated at 56°C for 30 min before use. Anti-spike IgG/IgA levels for variants in nasopharyngeal swabs and sera were measured using the V-PLEX SARS-CoV-2 panel 24 and 25 kits (Meso Scale Discovery), respectively, according to the manufacturer's instructions. The serum antibody titer for

ancestral nucleoprotein (N) was measured using an Elecsys anti-SARS-CoV-2 kit (Roche, Basel, Switzerland) according to the manufacturer's instructions. Concentrations of nasopharyngeal samples were determined from the electrochemiluminescence signals by back-fitting to the standard curve of S309 monomeric IgG/IgA and multiplying by the dilution factor, as described above. One arbitrary unit (AU) for ancestral spike IgG, IgA, BA.1 spike IgG, and IgA was converted to 0.421, 0.090, 5.653, and 0.599 ng, respectively.

Total IgG and IgA levels in nasopharyngeal swab fluids were measured using the Isotyping Panel 1 Human/NHP Kit (Meso Scale Discovery), according to the manufacturer's instructions. In **Fig. S2B-G**, anti-spike IgG, IgA, and S-IgA concentrations (ng/mL) were normalized by total IgG or IgA concentrations (ng/mL).

Mathematical modeling

To describe the SARS-CoV-2 infection dynamics in respiratory specimens, we used the following mathematical model developed in our recent studies (8-10):

$$\frac{df(t)}{dt} = -\beta f(t)V(t), \qquad (Eq. 1)$$

$$\frac{dV(t)}{dt} = \gamma f(t)V(t) - \delta V(t). \qquad (Eq. 2)$$

The variables $f(t)$ and $V(t)$ are the ratio of uninfected target cells and the amount of the virus, respectively, and the parameters β, γ, and δ are the rate constant for virus infection, maximum rate constant for viral replication, and death rate of infected cells, respectively.

Parameter estimation

A nonlinear mixed-effects modeling approach was used, which incorporated fixed effects, as well as random effects that described interpatient variability in parameters. Inclusion of random effects amounts to the partial pooling of the data from all patients to improve the parameter estimates applicable across cases. The parameter of patient k, θ_k $(= \theta \times e^{\pi_k})$, is a product of θ (the fixed effect) and π_k (the random effect), where π_k is assumed to follow a normal distribution: $N(0, \Omega)$. The fixed and random effects parameters were estimated using the stochastic approximation expectation/maximization (SAEM) algorithm and the empirical Bayes method, respectively (**Table S2**). The estimated individual parameters of individuals infected with the SARS-CoV-2 Omicron variant are shown in **Table S3**. A right-truncated normal distribution was used in the likelihood function to account for left censoring of the viral load data (when the viral load was not detectable) (11). MONOLIX 2019R2 (www.lixoft.com), a program for the maximum likelihood estimation of a nonlinear mixed-effects model, was used to fit the model to the viral load data. The initial values were changed multiple times to avoid a local minimum of the Akaike information criterion (AIC), and the robustness of the parameter estimation was confirmed. As time 0 in the original dataset is the day of symptom onset, the time when the infection started was also estimated along with other parameters, assuming $V(0) = 0.01$. We estimated the time from infection to symptom onset, assuming a specified viral load at the time of infection. We opted not to use the date of diagnosis as a criterion due to considerable individual variations in the days leading up to diagnosis.

Bayesian inference for post-diagnosis/onset parameters in the hybrid immunity

We estimated the L_{IgG}, L_{IgA}, $L_{\mathrm{S-IgA}}$, T_{ISO}, and T_{PCR} in immuno history groups using a Bayesian hierarchical model, based on a previous study (12). We inferred population means (μ_0) separately for parameters (Ps). We used a hierarchical structure to describe the distribution of μ_h for each immuno-history group. Arrays in the model index over one or more indices: H=4 immuno history *h*; N=122 participants *n*. The model was as follows:

$P_{hn} \sim Normal\ (\mu_h, \sigma_h)$
$\mu_h \sim Normal\ (\mu_0, \sigma_{\mu_0})$ [-5, 20]
$\mu_0 \sim Normal\ (10, 10)$ [-5, 20]
$\sigma_{\mu_0} \sim Student\_t\ (8, 0, 2.5)$ [0, ∞]
$\sigma_h \sim Student\_t\ (8, 0, 2.5)$ [0, ∞]

The values in square brackets denote the truncation bounds of the distributions. The explanatory variable was the outcome variable was P_{hn} in participant *n* in immuno history *h*. The mean parameter for Ps against according to the immuno history h, μ_{hv}, was generated from a normal distribution with hyperparameters of the mean, μ_h, and standard deviation, σ_h. For the distribution generating σ_h and σ_{μ_0}, we used a Student's t distribution with eight degrees of freedom, instead of a normal distribution, to reduce the effects of outlier values of σ_h and σ_{μ_0}. Parameter estimation was performed using a Markov chain Monte Carlo (MCMC) approach implemented in rstan 2.26.23 (https://mc-stan.org). Four independent MCMC chains were run with 5,000 steps in the warm-up and sampling iterations, with subsampling every five iterations. We confirmed that all estimated parameters showed <1.01 R-hat convergence diagnostic values and >500 effective sampling size values, indicating that the MCMC runs were convergent. The above analyses were performed using R 4.3.1 (https://www.r-project.org/).

Statistical analysis

Both univariate and multivariate generalized linear model logistic regressions were performed with the virus isolation test as the outcome, and viral load and IgG, IgA, and S-IgA as predictors. When necessary, multiple group comparisons were performed using an analysis of variance (ANOVA, for more than two numerical variables). Multiple comparisons were performed using the Tukey's method. Bonferroni correction was used for post-hoc analysis. The confidence intervals of continuous and categorical variables were calculated using *t* test and Wilson's test, respectively. Pearson's correlation coefficients were used to assess correlations between continuous variables. In the correlation matrix analyses, Spearman correlations between variables were calculated with false discovery rate (FDR) corrections, as described previously (13). In multiple regression analysis, each parameter was regressed against the parameters indicated in each figure. Interaction terms between the antibody parameters were not included. All statistical analyses were performed using the R software (version 4.2.0) and GraphPad Prism 9.3.1 (San Diego, CA, USA). Viral RNA loads and antibody titers below the detection limit were converted to detection limits. Viral titers in viral isolation-negative samples were converted to half the detection limit (24 $TCID_{50}$/mL) in **Fig. 1A** and **Fig. 4B**. Statistical significance was set at $p < 0.05$.

Supporting Figures

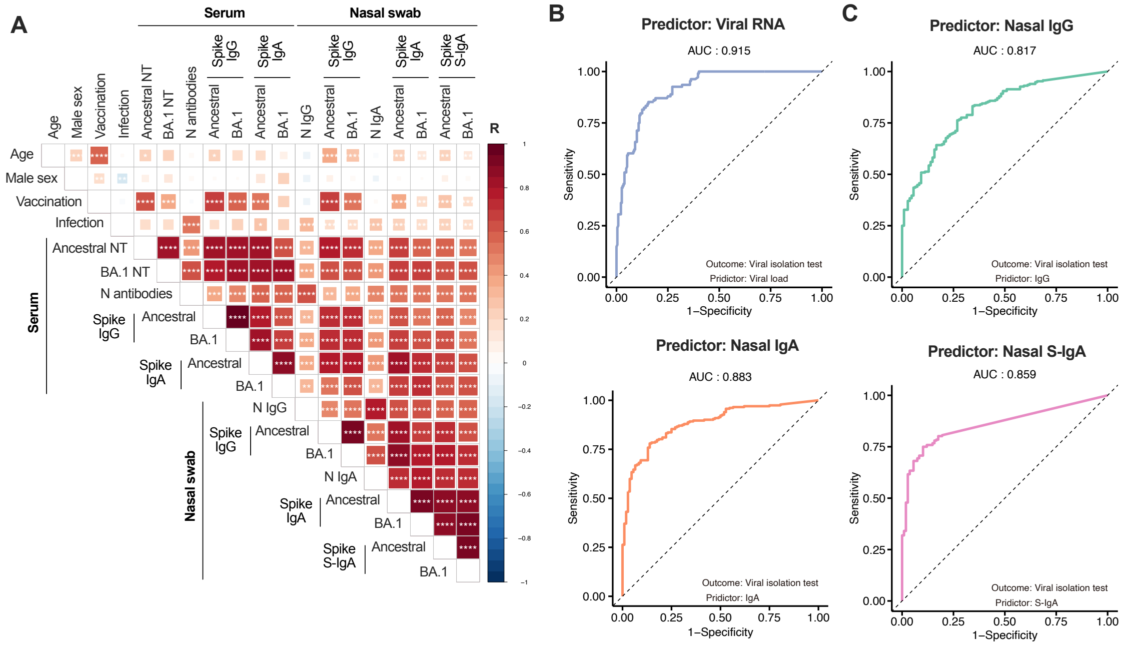

D

Threshold	Sensitivity (%)	Specificity (%)	AUC	Cut-off value
Viral RNA	85.2	84.5	0.915	5.364 ($\log_{10}$ copies/mL)
Nasal IgG	73.1	76.0	0.817	22.54 ng/mL
Nasal IgA	77.7	86.1	0.883	2.22 ng/mL
Nasal S-IgA	74.7	89.8	0.857	5.60 ng/mL

E

Threshold	Sensitivity (%)	Specificity (%)
Nasal IgG & viral RNA	64.0	95.3
Nasal IgA & viral RNA	74.1	94.8
Nasal S-IgA & viral RNA	76.9	94.0

F

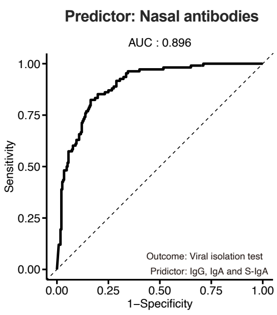

Fig. S1. Relationship between nasal antibody titers and serum antibody titers and prediction of infectious virus shedding. (A) Spearman correlation matrix of serum and nasal antibody titers with matching collection dates and case characteristics for Omicron-infected individuals. Male sex, vaccination status, and previous infection were used as dummy variables (1 vs. 0). Spearman's correlation R values were calculated using the square size and heat scale. The statistical significance level corrected using the false discovery rate (FDR) is shown as a square. Statistical significance: ns, not significant; *p < 0.05; **p < 0.01; ***p < 0.001; ****p < 0.0001. **(B, C, F)** ROC curve of generalized linear model logistic regression by viral RNA load **(B)**, individual nasal anti-BA.1 spike IgG, IgA, and S-IgA titers, and **(C)** nasal anti-BA.1 spike antibody titers **(F)** for predicting positive infectious virus shedding. The corresponding AUC-ROC is shown at the top of each panel. A summary of the logistic regression and calculated cut-off values is presented in **(D)**. **(E)** Summary of the sensitivity and specificity of the positive viral isolation test using a combination of thresholds.

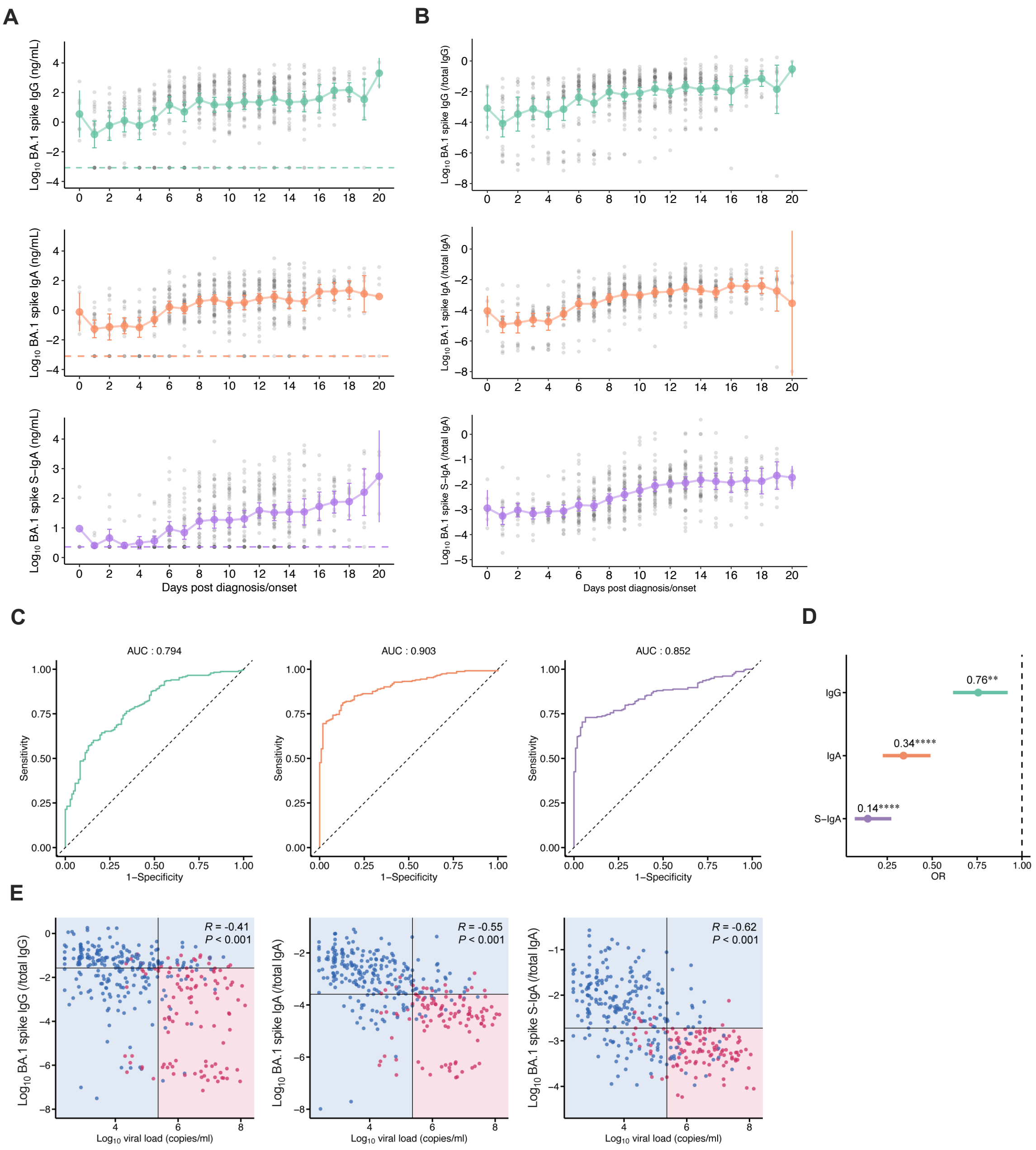

F

Threshold	Sensitivity (%)	Specificity (%)	AUC	Cut-off value (%)
Nasal IgG	60.1	84.3	0.794	2.63 (/total IgG)
Nasal IgA	81.1	87.0	0.903	0.03 (/total IgA)
Nasal S-IgA	73.0	93.5	0.852	0.19 (/total IgA)

G

Threshold	Sensitivity (%)	Specificity (%)
Nasal IgG & viral RNA	73.1	92.3
Nasal IgA & viral RNA	75.9	94.4
Nasal S-IgA & viral RNA	83.3	90.1

Fig. S2. Prediction of virus isolation by nasal anti-spike S-IgA titer against Omicron variants with adjustment by total IgA: (A) Dynamics of individual anti-spike antibodies against the Omicron BA.1 variant in nasal swabs in the days following diagnosis or onset of COVID-19 in SARS-CoV-2 Omicron-infected cases. Mean ± 95% confidence interval (CI) and individual data points are shown. Dotted lines indicate the detection limits. **(B)** Dynamics of individual anti-spike antibodies against the Omicron BA.1 variant in nasal swabs adjusted by indicated IgG or IgA in the Omicron-infected cases. Mean ± 95% confidence interval (CI) and individual data points are shown. **(C)** ROC curve of generalized linear model logistic regression by individual adjusted nasal anti-

BA.1 spike IgG, IgA, and S-IgA titers for predicting positive infectious virus shedding. **(D)** Logistic regression analysis of virus isolation-test positive samples, considering nasal anti-BA.1 spike antibody titers adjusted by indicated IgG or IgA. Forest plot showing odds ratio (OR) and 95% CI. **(E)** Verification of the accuracy of cut-off values estimated using logistic regressions. The areas and dots represent the predictive value and viral isolation test outcomes, respectively. Vertical and horizontal lines indicate cut-off values as shown in **Fig. S2E**. Pearson correlation R values, and p values are shown. **(F)** A summary of the logistic regression and calculated cut-off values. **(G)** Summary of the sensitivity and specificity of the positive viral isolation test using a combination of thresholds. Statistical significance: **p < 0.01; ****p < 0.0001.

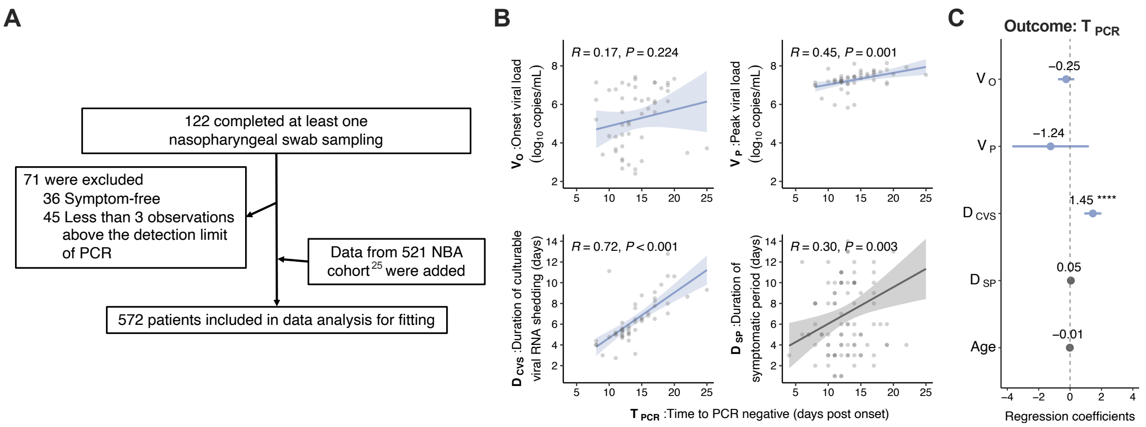

Fig. S3. Relationship between model-estimated and measured values. (A) Flowchart of the FF100 and NBA cohorts, along with the number of participants and inclusion criteria for our analysis. **(B)** Correlations between individual time to PCR-negative ($\mathrm{T_{PCR}}$) and onset viral load ($\mathrm{V_O}$), peak viral load ($\mathrm{V_P}$), cumulative viral load ($\mathrm{V_C}$), duration of culturable viral RNA shedding ($\mathrm{D_{CVS}}$), and duration of symptomatic period ($\mathrm{D_{SP}}$). Regression lines with 95% confidence intervals (CIs), Pearson correlation R-values and p values are shown. **(C)** Multiple regression analysis of T_{PCR}, including features extracted from the reconstructed viral RNA dynamics. Forest plot showing regression coefficients and 95% CI. Statistical significance: ****p < 0.0001.

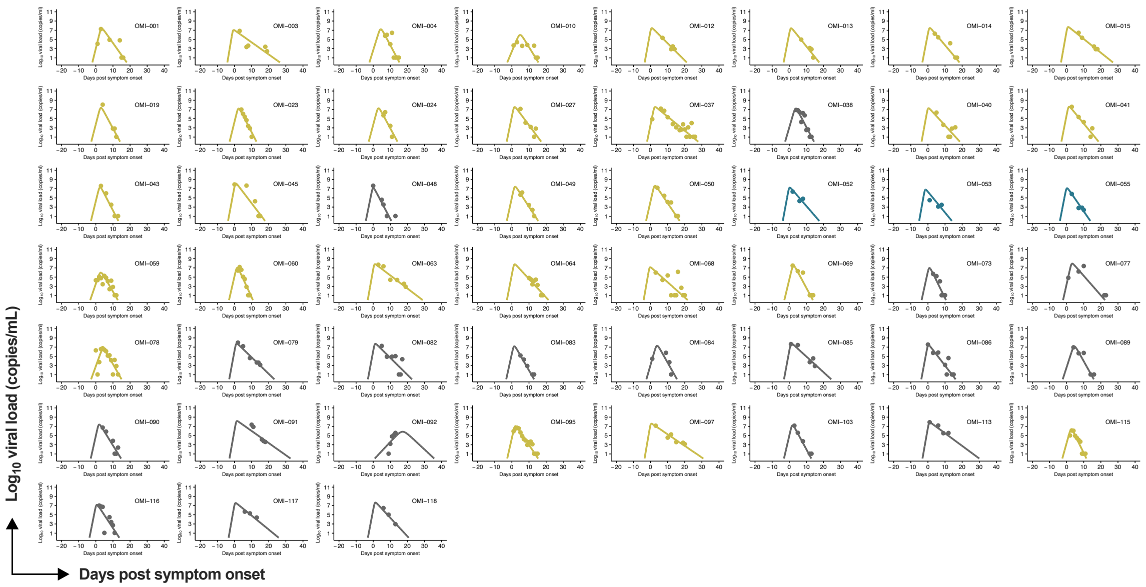

Fig. S4. Reconstructed viral dynamics for 51 individual participants from the FF100 cohort. The individual-level model fits the viral RNA loads in the respiratory samples using the model described in Eq. (1-2) for the cohorts described in **Fig. 2B**. The closed dots and solid curves indicate measured data and estimated viral dynamics, respectively. Individuals with different infection and vaccination histories are shown in different colors (gray, deep yellow, and deep blue for naïve, vaccination only, and prior infection, respectively).

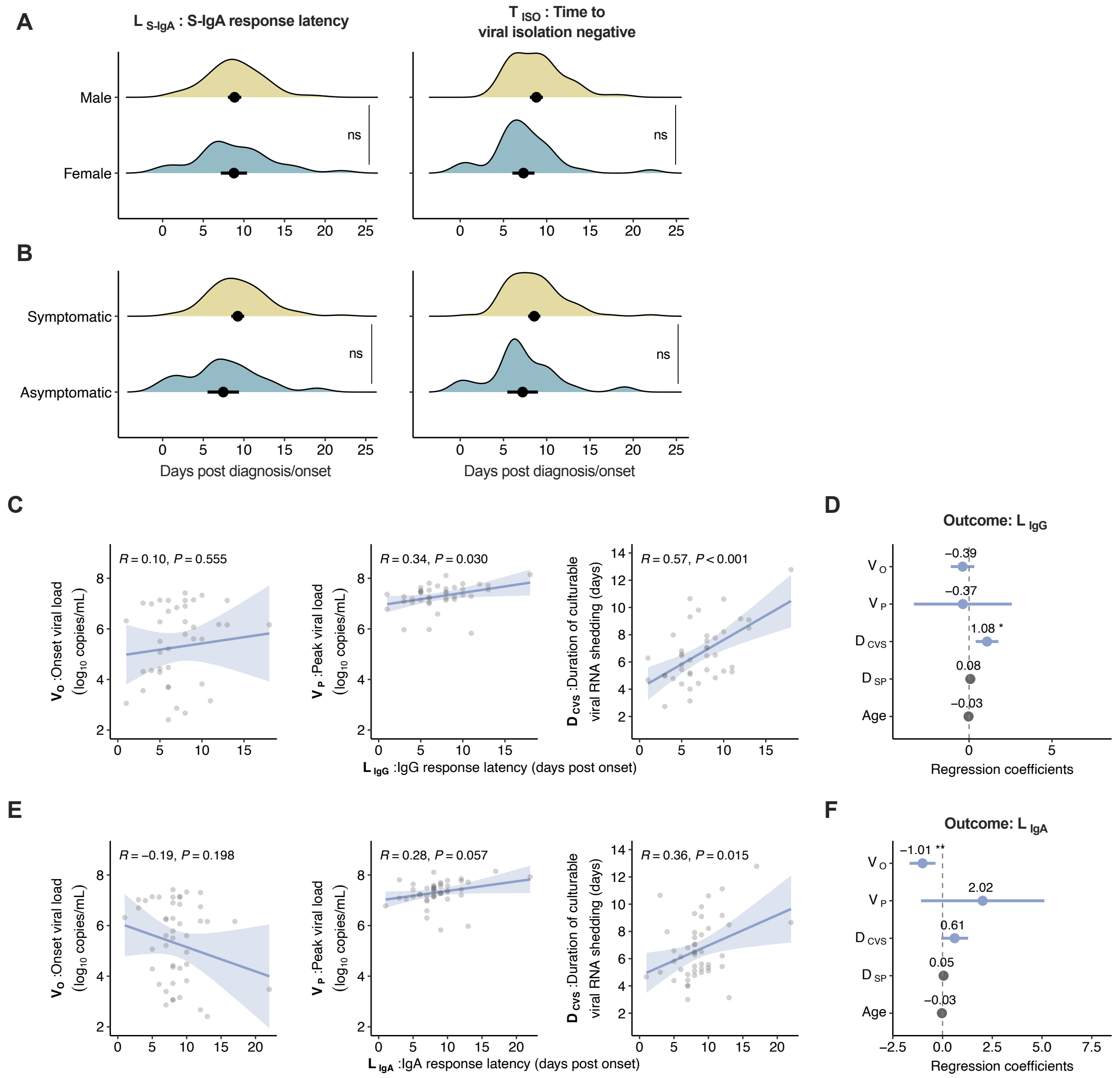

Fig. S5. Relationship between nasal anti-spike S-IgA response latency and case characteristics. Associations between individual S-IgA response latency ($\mathrm{L_{S-IgA}}$), time to isolation-negative ($\mathrm{T_{ISO}}$), **(A)** sex, and **(B)** symptom onset. The mean ± 95% CI is shown. Statistical significance was assessed using an unpaired t-test. **(C)** Correlations between post-onset IgG response latency ($\mathrm{L_{IgG}}$) and each of the following parameters: onset viral load ($\mathrm{V_O}$), peak viral load ($\mathrm{V_P}$), and duration of culturable viral RNA shedding ($\mathrm{D_{CVS}}$). **(D)** Multiple regression analysis of $\mathrm{L_{IgG}}$, including the features extracted from the reconstructed viral RNA dynamics. **(E)** Correlations between post-onset IgA response latency ($\mathrm{L_{IgA}}$) and each of the viral RNA features. **(F)** Multiple regression analysis of $\mathrm{L_{IgA}}$, including the viral RNA features. Regression lines with 95% CIs, Pearson correlation R values, and p values are shown. Forest plot showing regression coefficients and 95% CI. Statistical significance: *$p < 0.05$; **$p < 0.01$.

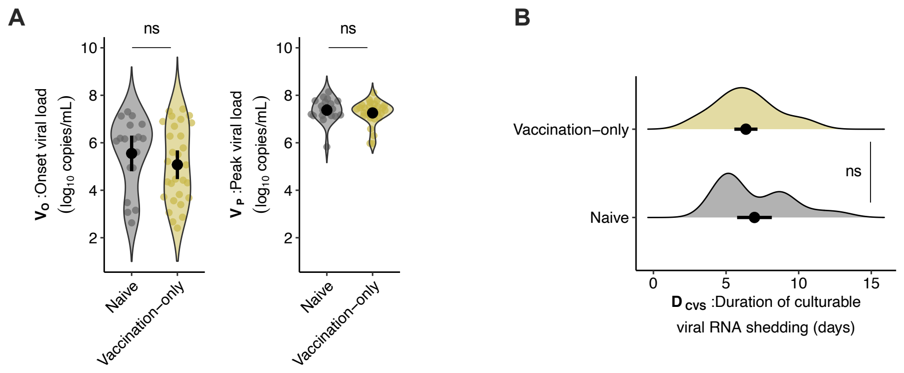

Fig. S6. Relationship between viral RNA shedding dynamics and vaccination status. Distribution of the **(A)** onset viral load (V_O), peak viral load (V_P), and **(B)** duration of culturable viral RNA shedding (D_{CVS}) according to immune history are shown. The mean ± 95% CI is shown. Statistical significance was assessed using an unpaired t-test. Statistical significance: ns, not significant.

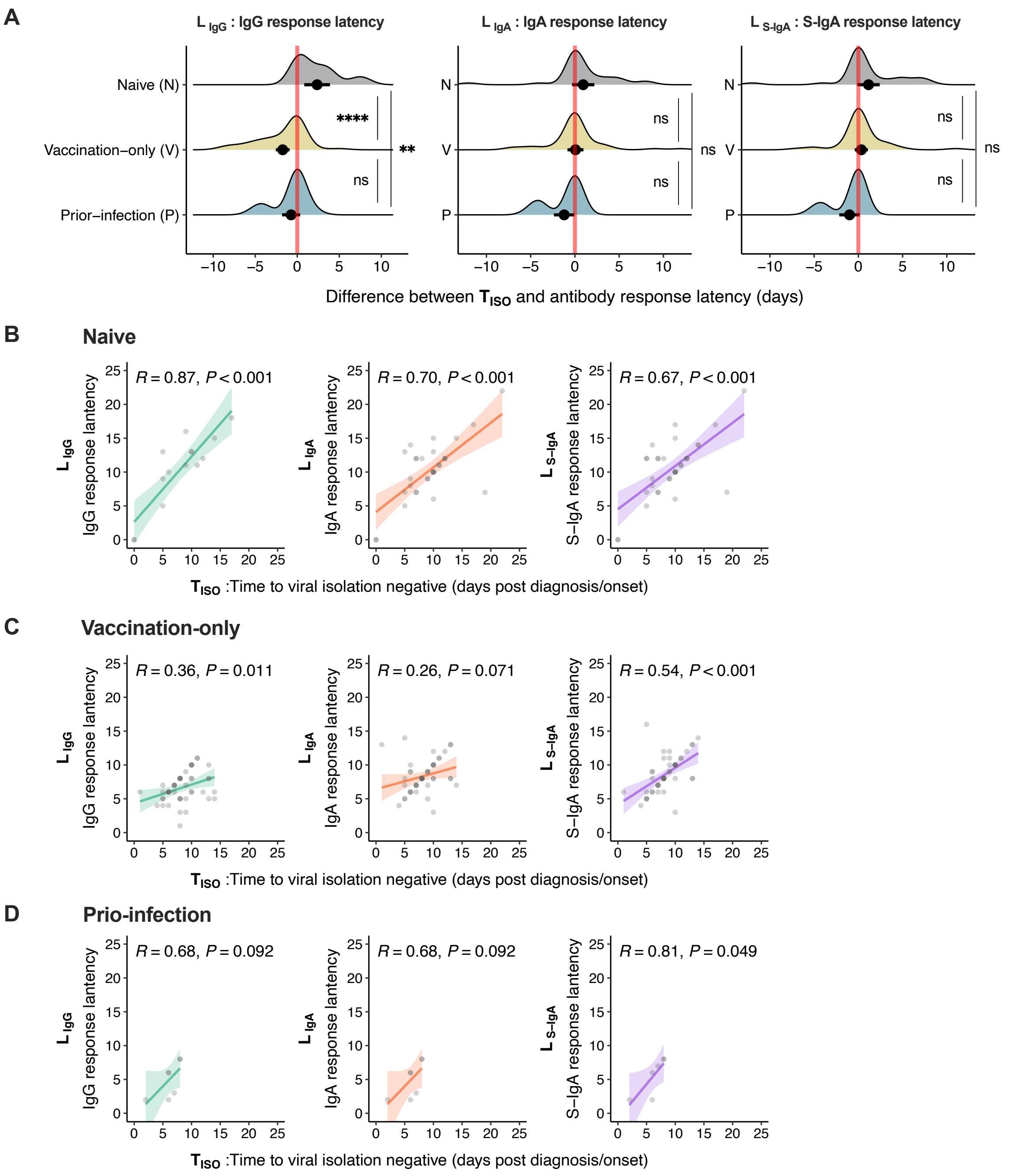

Fig. S7. Relationship among nasal anti-spike antibody response latency, virus shedding, and immuno histories: **(A)** Difference between time to isolation negative (T_{ISO}) and antibody response latency. Days on which the two features matched a difference of zero days) are highlighted in red. The mean ± 95% CI is shown. Significance was measured using one-way ANOVA and corrected using Tukey's test. **(B–D)** Correlations between T_{ISO} and each of the antibody response latencies after diagnosis/onset (L_{IgG}, L_{IgA}, and L_{S-IgA}) in Naïve (B), Vaccination-only (C), and Prior-infection (D) groups are shown. Regression lines with 95% CIs, Pearson correlation R values, and p values are shown. Statistical significance: ns, not significant; **p < 0.01; ***p < 0.001; ****p < 0.0001.

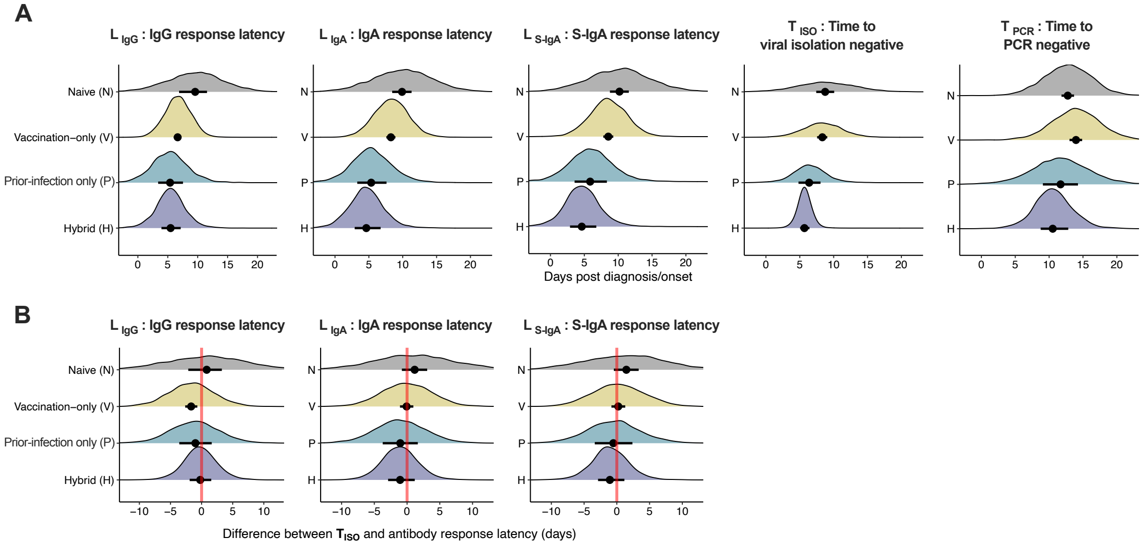

Fig. S8. Effect of Hybrid immunity on S-IgA response latency and viral shedding duration: **(A)** Bayesian inference of post-diagnosis/onset parameters obtained from the FF100 cohort among the exposure histories. **(B)** Difference between time to isolation negative (T_{ISO}) and antibody response latency. Days on which the two features matched (a difference of zero days) are highlighted in red. The predicted distribution (density) and the 95% credible interval for the mean (black bar) are shown.

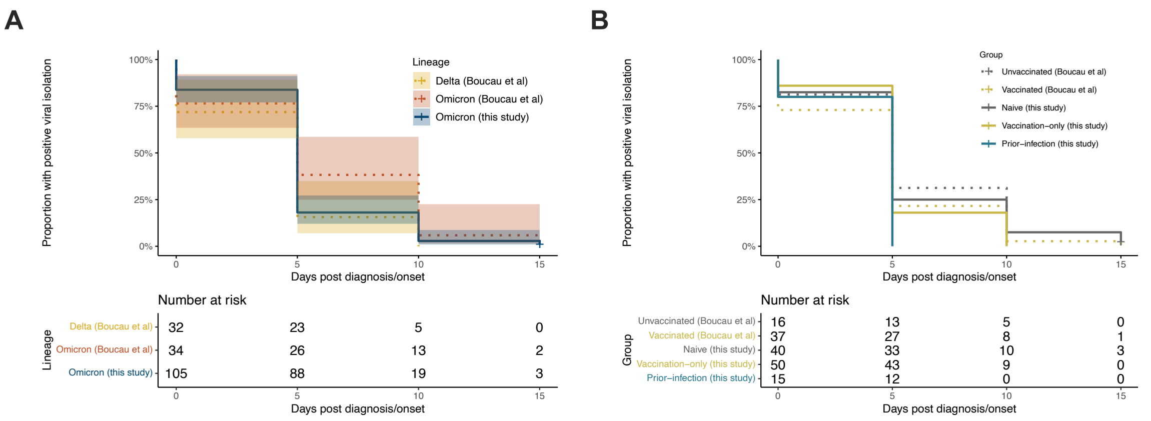

Fig. S9. Comparison of time to negative viral culture (i.e., T_{ISO}) between this study and the previous report by Boucau *et al.* (4): (A) Kaplan–Meier survival curves showing time from diagnosis/onset to negative viral culture categorized by viral variant. Shaded areas indicate 95% CIs. **(B)** Survival curves depicting time to negative viral culture based on immuno history.

Supporting Tables

Table S1. Case demographic characteristics and summary of the test results of the FF100 cohort

	**FF100 cohort**
**Number of cases**	122
**Age**, median (interquartile range)	31 (18, 45)
**Male sex**, N (%)	80 (66%)
**Vaccine dose count**, N (%)	
No dose	60 (49%)
1 dose	3 (2.5%)
2 doses	54 (44%)
3 doses	5 (4.1%)
**Vaccine type**, N (%)	
BNT162b2	29 (24%)
mRNA-1273	21 (17%)
AZD1222	6 (4.9%)
Ad26.COV2.S	2 (1.6%)
Combined	3 (2.5%)
Not listed	61 (50%)
**Severity**, N (%)	
Asymptomatic	26 (21%)
Mild	90 (74%)
Moderate	5 (4.1%)
Severe	1 (0.8%)
**Infection lineage**, N(%)	
BA.1	110 (90.2%)
BA.2	5 (4.1%)
Omicron confirmed (BA.1 or BA.2)	7 (5.7%)
**Number of nasopharyngeal swabs**	590
**Viral isolation test**, N (%; 95% CI)	
Positive	108 (18.3%; 15.3, 21.7%)
Negative	444 (75.3%; 71.5, 78.6%)
N/A	38 (6.4%; 4.7, 8.8%)
**Viral RNA load**, Mean (95% CI) (log_{10} copies/mL)	3.99 (3.85, 4.12)
**Viral titer**, Mean (95% CI) (log_{10} $TCID_{50}$/mL)	1.36 (1.30, 1.42)
**Anti-ancestral antibody titer**, Mean (95% CI)	
Anti-spike IgG (log_{10} ng/mL)	0.41 (0.25, 0.56)
Anti-spike IgA (log_{10} ng/mL)	-0.08 (-0.19, 0.03)
Anti-spike S-IgA (log_{10} ng/mL)	0.88 (0.82, 0.95)
**Anti-BA.1 antibody titer**, Mean (95% CI)	
Anti-spike IgG (log_{10} ng/mL)	1.10 (0.95, 1.24)
Anti-spike IgA (log_{10} ng/mL)	0.37 (0.25, 0.49)
Anti-spike S-IgA (log_{10} ng/mL)	1.22 (1.14, 1.29)
**Number of sera**	86
**Anti-ancestral antibody titer**, Mean (95% CI)	
Anti-spike IgG (log_{10} AU/mL)	4.95 (4.67, 5.23)
Anti-spike IgA (log_{10} AU/mL)	3.70 (3.50, 3.90)
Neutralization titer (log_{10})	1.75 (1.53, 1.96)
**Anti-BA.1 antibody titer**, Mean (95% CI)	
Anti-spike IgG (log_{10} AU/mL)	4.47 (4.21, 4.73)
Anti-spike IgA (log_{10} AU/mL)	3.40 (3.24, 3.57)
Neutralization titer (log_{10})	1.66 (1.48, 1.83)

Table S2. Estimated fixed and random effects for SARS-CoV-2 Omicron variants

Parameters	Description	Unit	$\boldsymbol{\vartheta}$: Fixed effect (SE)*	$\boldsymbol{\Omega}$: SD of random effect (SE)*
β	Rate constant for virus infection	(RNA copies/ml)$^{-1}$day^{-1}	1.36×10^{-7} (1.07×10^{-8})	1.33 (0.070)
γ	Maximum rate constant for viral replication	Day^{-1}	5.6 (0.06)	0.13 (0.001)
δ	Death rate of virus-producing cells	Day^{-1}	1.24 (0.04)	0.53 (0.022)
τ	Days from infection to symptom onset	Days	4.58 (0.09)	0.28 (0.013)

* The parameter for patient k, ϑ_i $(= \vartheta \times e^{\pi_k})$, is represented as a product of ϑ (a fixed effect) and e^{π_k} (a random effect). π_k follows the normal distribution with mean 0 and standard deviation Ω. SE: standard error.

Table S3. Estimated individual parameters for SARS-CoV-2 Omicron variants.

Parameter	Rate constant for virus infection	Maximum rate constant for viral replication	Death rate of virus-producing cells	Days from infection to symptom onset
**Symbol**	β	γ	δ	τ
**Unit**	$(\text{RNA copies/ml})^{-1}\text{day}^{-1}$	Day^{-1}	Day^{-1}	Day
**Individual estimated parameters**				
OMI-001	0.0074	4.82	1.36	3.39
OMI-003	0.0332	5.67	0.61	5.09
OMI-004	0.0536	5.42	2.67	3.90
OMI-010	0.0520	5.00	2.95	4.97
OMI-012	0.0106	5.55	0.87	4.42
OMI-013	0.0098	5.51	1.11	4.34
OMI-014	0.0090	5.49	1.05	4.30
OMI-015	0.0068	5.50	0.69	4.24
OMI-019	0.0060	5.47	2.16	4.04
OMI-023	0.0088	5.74	2.28	4.21
OMI-024	0.0070	5.41	2.19	4.05
OMI-027	0.0071	5.40	1.22	4.13
OMI-037	0.0083	4.90	0.71	3.13
OMI-038	0.0071	5.47	2.54	3.97
OMI-040	0.0162	5.62	0.95	4.71
OMI-041	0.0067	5.42	1.08	4.13
OMI-043	0.0073	5.69	2.21	4.18
OMI-045	0.0021	5.72	1.17	4.79
OMI-048	0.0144	6.14	1.92	5.40
OMI-049	0.0077	5.40	1.47	4.14
OMI-050	0.0044	5.17	1.41	3.84
OMI-052	0.0175	5.64	1.01	4.77
OMI-053	0.0489	5.85	1.07	6.09
OMI-055	0.0203	5.72	1.32	4.98
OMI-059	0.0820	5.01	2.52	5.13
OMI-060	0.0140	6.31	2.58	4.45
OMI-063	0.0053	5.48	0.65	4.16
OMI-064	0.0046	5.35	0.95	3.97
OMI-068	0.0247	5.67	0.77	4.98
OMI-069	0.0051	5.59	2.04	4.17
OMI-073	0.0176	6.14	2.24	4.88
OMI-077	0.0026	4.84	1.02	3.27
OMI-078	0.0167	5.24	2.87	4.72
OMI-079	0.0053	5.44	0.83	4.10
OMI-082	0.0058	5.43	0.86	4.11
OMI-083	0.0125	5.65	1.76	4.49
OMI-084	0.0057	5.14	2.17	3.99
OMI-085	0.0053	5.42	0.79	4.06
OMI-086	0.0102	5.72	1.15	4.88
OMI-089	0.0064	5.16	2.51	4.03
OMI-090	0.0080	5.42	1.58	4.16
OMI-091	0.0025	5.40	0.61	3.92
OMI-092	0.0129	5.12	4.20	4.15
OMI-095	0.0172	5.19	1.43	3.95
OMI-097	0.0099	5.57	0.58	4.44
OMI-103	0.0101	5.66	1.97	4.34
OMI-113	0.0049	5.49	0.63	4.15
OMI-115	0.0503	5.89	2.81	4.02
OMI-116	0.0165	5.70	1.40	4.80
OMI-117	0.0097	5.55	0.70	4.40
OMI-118	0.0065	5.43	0.94	4.14

Table S4. Summary of the features of viral RNA dynamics for two cohorts

**Feature**	**Units**	**FF100,** mean (95% CI)	**NBA,** mean (95% CI)
Onset viral load	Log_{10} copies/mL	5.24 (4.75-5.73)	5.65 (5.52-5.78)
Peak viral load	Log_{10} copies/mL	7.29 (7.15-7.43)	7.21 (7.17-7.25)
Virus expansion rate	Day^{-1}	3.94 (3.70-4.19)	4.15 (4.08-4.21)
Virus clearance rate	Day^{-1}	1.54 (1.31-1.77)	1.44 (1.38-1.50)
Duration of culturable viral RNA shedding	Days	6.52 (5.90-7.14)	6.14 (5.95-6.32)

Table S5. Case demographic characteristics of the S-IgA response latency groups

Group	Long latency (L_{SIgA} >5)		Short latency (L_{SIgA} ≤5)	
	N (%); Median	95%CI	N (%); Median	95%CI
**Number of cases**	88		15	
**Age**	33.5	30, 38	30.0	18, 41
**Male sex**	59 (67%)	56%, 77%	9 (60%)	32%, 84%
**Vaccine dose count**				
No dose	40 (46%)	35%, 56%	6 (40%)	16%, 68%
1 dose	3 (3.4%)	0.7%, 9.6%	0 (0.0%)	0%, 22%
2 doses	41 (47%)	36%, 58%	9 (60%)	32%, 84%
3 doses	4 (4.5%)	1.3%, 11%	0 (0.0%)	0%, 22%
**Vaccine type**				
BNT162b2	21 (24%)	15%, 34%	6 (40%)	16%, 68%
mRNA-1273	18 (21%)	13%, 30%	1 (6.7%)	0.2%, 32%
AZD1222	4 (4.5%)	1.3%, 11%	2 (13%)	1.7%, 41%
Ad26.COV2.S	1 (1.1%)	0.0%, 6.2%	0 (0.0%)	0%, 22%
Combined	3 (3.4%)	0.7%, 9.6%	0 (0.0%)	0%, 22%
Not listed	41 (47%)	36%, 58%	6 (40%)	16%, 68%
**Severity**				
Asymptomatic	18 (21%)	13%, 30%	6 (40%)	16%, 68%
Mild	65 (74%)	63%, 83%	9 (60%)	32%, 84%
Moderate	4 (4.5%)	1.3%, 11%	0 (0.0%)	0%, 22%
Severe	1 (1.1%)	0.0%, 6.2%	0 (0.0%)	0%, 22%
**Prior infection**	7 (8.0%)	3.2%, 15.7%	6 (40%)	16.3%, 67.7%
**Days from last exposure to diagnosis**	124	112, 151	109	60, 213
N (%)	47 (53%)		9 (60%)	
**Days from last exposure to onset**	129	117, 152	107	34, 172
N (%)	40 (45%)		6 (40%)	
**Days from onset to diagnosis**	-1.0	-1.1, -0.1	0.0	-0.7, 0.3
N (%)	70 (79%)		9 (60%)	
**Days from infection to onset**	4.2	4.1, 4.4	4.8	4.1, 5.7
N (%)	43 (49%)		6 (40%)	

Table S6. Case demographic characteristics and summary of the test results of nasopharyngeal samples of the immune history groups

Group	Naïve	Vaccination-only	Prior-infection
**Number of cases**	53	54	15
**Age***	16 (6, 28)	40 (30, 49)	37 (23, 46)
**Male sex**, N (%)	34 (64%)	39 (72%)	7 (47%)
**Vaccine dose count**, N (%)			
No dose	53 (100%)	0 (0.0%)	7 (47%)
1 dose	0 (0.0%)	2 (3.7%)	1 (6.7%)
2 doses	0 (0.0%)	47 (87%)	7 (47%)
3 doses	0 (0.0%)	5 (9.2%)	0 (0.0%)
**Vaccine type**, N (%)			
BNT162b2	0 (0.0%)	25 (46%)	4 (27%)
mRNA-1273	0 (0.0%)	20 (37%)	1 (6.6%)
AZD1222	0 (0.0%)	3 (5.5%)	3 (20%)
Ad26.COV2.S	0 (0.0%)	2 (3.7%)	0 (0.0%)
Combined	0 (0.0%)	3 (5.5%)	0 (0.0%)
Not listed	53 (100%)	1 (1.8%)	7 (47%)
**Severity**, N (%)			
Asymptomatic	13 (25%)	7 (13%)	6 (40%)
Mild	38 (72%)	43 (80%)	9 (60%)
Moderate	1 (1.9%)	4 (7.4%)	0 (0.0%)
Severe	1 (1.9%)	0 (0.0%)	0 (0.0%)
**Timing of prior-infection**, N (%)			
Before first vaccination	N/A	N/A	2 (13%)
After first vaccination	N/A	N/A	0 (0.0%)
Not listed	N/A	N/A	13 (87%)
**Period of prior-infection**	N/A	N/A	Dec.2020 – Aug.2021
**Days from last exposure to diagnosis***	N/A	117 (104, 141)	154 (101, 223)
N (%)	N/A	49 (91%)	4 (27%)
**Days from last exposure to onset***	N/A	121 (107, 151)	129 (99, 168)
N (%)	N/A	43 (80%)	6 (40%)
**Days from onset to diagnosis***	-1 (-1, 0)	0 (-1, 1)	0 (-1, 0)
N (%)	40 (75%)	47 (87%)	9 (60%)
**Days from infection to onset***	4.2 (4.1, 4.5)	4.2 (4.0, 4.5)	5.0 (4.9, 5.5)
N (%)	19 (35%)	29 (54%)	3 (20%)
**Number of nasopharyngeal samples**	220	315	55
**Viral isolation test**, N (%; 95% CI)			
Positive	46 (21%; 16, 27%)	56 (18%; 14, 23%)	6 (11%; 4.5, 23%)
Negative	145 (66%; 59, 72%)	250 (79%; 74, 84%)	49 (89%; 77, 96%)
N/A	29 (13%; 9.2, 19%)	9 (2.9%; 1.4, 5.5%)	0 (0%; 0.0, 8.1%)
**Viral RNA load**, Mean (95% CI) (log_{10} copies/mL)	4.06 (3.83, 4.29)	4.04 (3.85, 4.22)	3.43 (3.04, 3.81)
**Viral titer**, Mean (95% CI) (log_{10} $TCID_{50}$/mL)	1.46 (1.35, 1.57)	1.31 (1.24, 1.38)	1.28 (1.06, 1.49)

**Anti-ancestral antibody titer**, Mean (95% CI)			
Anti-spike IgG (log_{10} ng/mL)	-1.32 (-1.51, -1.14)	1.45 (1.31, 1.59)	1.32 (0.96, 1.67)
Anti-spike IgA (log_{10} ng/mL)	-0.84 (-0.98, -0.69)	0.29 (0.15, 0.44)	0.80 (1.46, 1.15)
Anti-spike S-IgA (log_{10} ng/mL)	0.52 (0.45, 0.58)	1.05 (0.95, 1.14)	1.43 (1.22, 1.65)
**Anti-BA.1 antibody titer**, Mean (95% CI)			
Anti-spike IgG (log_{10} ng/mL)	-0.11 (-0.35, 0.12)	1.80 (1.63, 1.96)	1.92 (1.56, 2.29)
Anti-spike IgA (log_{10} ng/mL)	-0.08 (-0.27, 0.11)	0.53 (0.37, 0.69)	1.20 (0.92, 1.48)
Anti-spike S-IgA (log_{10} ng/mL)	0.96 (0.84, 1.07)	1.32 (1.21, 1.43)	1.67 (1.43, 1.92)

* median (interquartile range)

SI References

1. K. Takahashi *et al.*, Duration of Infectious Virus Shedding by SARS-CoV-2 Omicron Variant-Infected Vaccinees. *Emerg Infect Dis* **28**, 998-1001 (2022).
2. K. Shirato *et al.*, Development of Genetic Diagnostic Methods for Detection for Novel Coronavirus 2019(nCoV-2019) in Japan. *Jpn J Infect Dis* **73**, 304-307 (2020).
3. S. M. Kissler *et al.*, Viral dynamics of acute SARS-CoV-2 infection and applications to diagnostic and public health strategies. *PLoS Biol* **19**, e3001333 (2021).
4. J. Boucau *et al.*, Duration of Shedding of Culturable Virus in SARS-CoV-2 Omicron (BA.1) Infection. *N Engl J Med* **387**, 275-277 (2022).
5. S. Miyamoto *et al.*, Vaccination-infection interval determines cross-neutralization potency to SARS-CoV-2 Omicron after breakthrough infection by other variants. *Med* **3**, 249-261.e244 (2022).
6. D. Pinto *et al.*, Cross-neutralization of SARS-CoV-2 by a human monoclonal SARS-CoV antibody. *Nature* **583**, 290-295 (2020).
7. S. Saito *et al.*, IgA tetramerization improves target breadth but not peak potency of functionality of anti-influenza virus broadly neutralizing antibody. *PLOS Pathogens* **15**, e1007427 (2019).
8. S. Iwanami *et al.*, Detection of significant antiviral drug effects on COVID-19 with reasonable sample sizes in randomized controlled trials: A modeling study. *PLoS Med* **18**, e1003660 (2021).
9. Y. D. Jeong *et al.*, Designing isolation guidelines for COVID-19 patients with rapid antigen tests. *Nat Commun* **13**, 4910 (2022).
10. K. S. Kim *et al.*, A quantitative model used to compare within-host SARS-CoV-2, MERS-CoV, and SARS-CoV dynamics provides insights into the pathogenesis and treatment of SARS-CoV-2. *PLoS Biol* **19**, e3001128 (2021).
11. A. Samson, M. Lavielle, F. Mentré, Extension of the SAEM algorithm to left-censored data in nonlinear mixed-effects model: Application to HIV dynamics model. *Comput Stat Data Anal* **51**, 1562-1574 (2006).
12. S. Miyamoto *et al.*, Saturation time of exposure interval for cross-neutralization response to SARS-CoV-2: Implications for vaccine dose interval. *iScience* **26**, 106694 (2023).
13. S. Miyamoto *et al.*, Non-Omicron breakthrough infection with higher viral load and longer vaccination-infection interval improves SARS-CoV-2 BA.4/5 neutralization. *iScience* **26**, 105969 (2023).
